# Relational and symbolic process: decisions regarding the place of death in the context of advanced cancer*

**DOI:** 10.1590/1980-220X-REEUSP-2025-0405en

**Published:** 2026-07-31

**Authors:** Patrícia Chatalov Ferreira, Marcelle Miranda da Silva, Mayckel da Silva Barreto, Esperanza Begoña Garcia-Navarro, Cristina Garcia-Vivar, Eleandro do Prado, Rudval Souza da Silva, Sonia Silva Marcon

**Affiliations:** 1 Universidade Estadual de Maringá, Programa de Pós-Graduação em Enfermagem, Maringá, PR, Brazil.; 2 Universidade Federal do Rio de Janeiro, Programa de Pós-Graduação em Enfermagem, Rio de Janeiro, RJ, Brazil.; 3 Universidade de Huelva, Departamento de Enfermagem, Huelva, España.; 4 Universidad Pública de Navarra, Departamento de Enfermagem, Pamplona, Navarra, España.; 5 Universidade Positivo, Colegiado de Enfermagem, Curitiba, PR, Brazil.; 6 Universidade do Estado da Bahia, Programa de Pós-Graduação em Ciências do Cuidar em Saúde, Senhor do Bonfim, BA, Brazil.

**Keywords:** Palliative Care, Neoplasms, Decision Making, Family, Nursing, Grounded Theory.

## Abstract

**Objective:**

To construct a substantive theory regarding the meanings attributed to and the factors influencing the decision regarding the place of death in the context of advanced cancer.

**Methodology:**

Symbolic Interactionism and Grounded Theory were used as theoretical frameworks. Audio-recorded interviews, conducted in person or remotely between 2023 and 2024, were carried out with 96 participants from nine states located in four Brazilian regions, who were organized into five sample groups.

**Results:**

The hospital is perceived as a space offering protection, relief from pain and suffering, and relief from family burden, while the home is seen as an environment that promotes the patient’s dignity and autonomy and fosters emotional connection with family members, accompanied by uncertainty regarding the management of pain and suffering. Professional mediation and active listening facilitate the negotiation of meanings, while structural and communicational barriers limit the feasibility of choices.

**Final Considerations:**

The decision regarding the place of death constitutes a symbolic, relational, and dynamic process, shaped by family conflicts, limitations in the physical structure, and interactions among patients, family members, and the multidisciplinary team. It is important to implement public policies that enable choices consistent with the values of the person with a terminal illness.

## INTRODUCTION

The decision regarding the place of death in the context of patients with advanced cancer has become a central issue, especially given the progressive increase in neoplasms and complex chronic conditions, which intensifies challenges for health systems in developing countries, such as Brazil^(1)^. The COVID-19 pandemic has exacerbated this reality, delaying diagnoses and compromising access to services, which has contributed to the late identification of advanced-stage cases^(2)^.

Coping with an irreversible condition, although it does not imply immediate death, requires complex decisions related to end-of-life care. Among these, the following stand out: determining the setting in which one wishes to die, choosing the location for palliative care, and organizin^(3)^

The pragmatic notion of a “good enough death” focuses on providing adequate care and managing physical, emotional, and spiritual symptoms. This shift would explain the divergence between the hardly attainable expectations of an idealized vision of death and the everyday reality of work in these services, marked by complexity and heterogeneity^(4)^. Thus, decisions regarding the place of death are influenced by clinical, emotional, social, and symbolic factors, which explains the diversity of preferences and motivations guiding these choices^(5)^. Therefore, understanding this decision-making process requires considering not only objective aspects but also the meanings constructed through socia

Most studies addressing the choice of place of death have been conducted in developed countries, presenting narrow approaches that give little consideration to the interactive nature of the decision-making process^(3,5,6)^. This gap becomes even more relevant when observed in the Brazilian context, marked by inequalities in access to health services and cultural diversity—factors that make this debate even more complex^(3,7)^.

In the national and international literature, quantitative studies link the choice of place of death to sociodemographic characteristics, such as age, marital status, and family support^(3,5,6,7,8)^. However, qualitative investigations that explore in depth the meanings attributed and the social interactions underpinning this process remain scarce^(9)^. This lack of studies not only hinders a broader and more contextualized understanding of the phenomenon but also limits the development of theoretical and practical frameworks capable of guiding policies and professional practices that are more sensitive to the reality experienced by people. In Brazil, this scarcity is even more evident in nursing journals, reinforcing the need for studies that broaden the understanding of this phenomenon from an interactional and practica^(7)^

In the national context, research on the decision-making process regarding the place of death remains incipient and fragmented, especially when considering the relational complexity involving the triad of patients, family members, and healthcare teams. The originality of this study lies in the comprehensive and interactional approach adopted, which prioritizes the construction of shared meanings and the communicational dynamics involved in this process. By proposing a substantive theory grounded in empirical evidence, this study broadens the understanding of the phenomenon beyond individual variables, offering an innovative perspective that integrates subjective, social, and institutional dimensions of end-of-life care^(7)^.

Furthermore, it is worth noting that this research is also aligned with the guidelines of the National Palliative Care Policy (PNCP in the Portuguese acronym)^(10)^, which emphasizes the centrality of the person and the family in care decisions, as well as the need to expand equitable access to quality services at all levels of care, especially in a country like Brazil, where rankings on the quality of palliative care place it 79th out of 81 countries^(11)^. Understanding the factors and meanings that guide decisions regarding the place of death directly contributes to the implementation of this policy by supporting more humanized, communicative, and culturally sensitive care practices. Furthermore, it can be assumed that the findings will support managers and professionals in formulating strategies aimed at organizing care networks, strengthening palliative care as an essential component of the health system, and reaffirming the role of nursing in the coordination and comprehensiveness of end-of-lif

Given this gap, the objective of the study was to construct a substantive theory regarding the meanings attributed and the factors influencing the decision regarding the place of death in the context of advanced cancer. Such understanding has direct implications for the practice of oncology nursing and for the organization of palliative care services, by promoting care strategies that are more sensitive to the needs of people with advanced cancer and thei

## METHOD

### Study Design and Theoretical and Methodological Framework

This is a qualitative, explanatory study that used Symbolic Interactionism (SI) as its theoretical framework, according to which the meanings attributed to situations or things emerge from social interactions and lived experiences^(12)^. Gounded Theory (GT), a constructivist approach, was the methodological choice. This approach assumes that the experiences of both the researcher and the participants are relevant and influence the interpretation of the data and, consequently, the construction of the phenomenon^(13)^. The research report was prepared in accordance with the *Consolidated Criteria for Reporting Qualitative Research* COREQ) guidelines.

### Location, Population and Selection Criteria

A total of 96 people participated in the study, distributed across five sample groups (SG) consisting of relatives of people who had died of cancer within the past year, people with advanced cancer, physicians, psychologists, and social workers, and finally nurses and physica

The inclusion criteria for patients and family members were being 18 years of age or older and having the ability to communicate verbally. Individuals with emotional instability or without access to technological resources—in the case of remote interviews—were not included. For professionals, the requirement was at least six months of experience in caring for people wit

### Theoretical Sampling

Data collection began with four family members of individuals who had died from cancer, referred by professionals from a charitable organization operating in the municipality where the study was conducted. At the end of the interviews, participants were asked to refer new potential participants, as proposed by the non-probabilistic sampling technique known as *Snowball Sampling* ^(14)^. However, there were difficulties in securing the participation of those referred, and considering that social media has been used satisfactorily as a setting for qualitative studies, including in addressing sensitive topics such as domestic violence^(15)^, we opted to explore other cultural and socioeconomic contexts, recruiting family members and people with advanced cancer who shared their experiences using social media as

Studies included in a review^(16)^ indicate that recruiting participants through social media is an effective and low-cost strategy in health research, provided it is accompanied by rigorous credibility verification criteria, such as preliminary screening, cross-checking of information, and mechanisms for identifying inappropriat

Thus, in an effort to mitigate ethical risks, the selection of these participants was conducted with great care, considering not only the aspects mentioned above but also mediation by group administrators and confirmation of eligibility during initial contact, in accordance with methodological recommendations for remote collection of qualitative data^(17)^. Furthermore, the ethical risks inherent in this type of recruitment were mitigated by obtaining informed consent, ensuring confidentiality, respecting participants’ privacy, and continuously monitoring for potential emotional distress, in line with the specialize^(18)^

The first sample group (SG) consisted of 15 family members of individuals who had died from cancer (five in face-to-face interviews and 10 in online interviews). Analysis of this group’s data showed that the decision regarding the place of death is influenced by family dynamics and communication with healthcare professionals, marked by the “Circle of Silence” and a “Paternalistic Model.” This led to the hypothesis that the “protective” strategies adopted by family members may interfere with the autonomy of people with advanced cancer (PAC) regarding the decision about the place of death. Thus, it was deemed necessary to include these individuals and hear their perspectives regarding autonomy in choosing the place o

Thus, the second focus group consisted of 24 PACs, identified through Instagram profiles that mentioned terms such as “advanced cancer,” “metastasis,” “metastatic,” “palliative,” and “palliative care” in their bios or public posts. Invitations to participate were sent via direct messages, and once accepted, a date and time were scheduled for the interview via video call on Google Meet. The guiding question for this group was “Tell me your story with cancer.” The topic of choosing a place of death was only broached at an appropriate moment, after the researcher sensed openness to the subject, so as not to trigger emotiona

During the collection and analysis of the interviews, it was found that the PAC expressed a preference regarding the place of death; however, it was noted that there were people who did not have the space to discuss this with their family members or healthcare professionals, especially with their doctor. This context raised the following question: What is the opinion of physicians regarding the choice of place of death for PAC? The hypothesis was that physicians believe that PAC is not capable of making thi

Thus, the third focus group consisted of 20 physicians. The guiding question was: “Tell me about the decisions regarding the place of death for PAC that you are currently involved in or have been involved in.” Analysis of this group’s data showed that oncologists and palliative care specialists address issues regarding death, palliative care, and place of death in diverse ways with family members and with the PCP themselves. This led to the following question: What is the role of other healthcare professionals in the decision-making process? The hypothesis was that psychological support for patients and family members influenced acceptance of terminal illness and the decision regarding the place o

Consequently, the fourth focus group consisted of 15 professionals, including 11 psychologists and four social workers. It was observed that the former assist physicians in difficult communication and work directly with the emotional and psychological dimensions of terminal illness, while social workers proved to be fundamental in supporting family members by handling logistical, social, and financia

Based on the data obtained from this group, it was observed that social and economic factors strongly influence the decision regarding the place of death and that logistical support enables end-of-life care at home. Furthermore, some reports from PACs and family members highlighted that nurses are directly involved in providing care and emotional support, both in the hospital and at home, in addition to frequently mediating between doctors and family members/patients, and that physical therapists assisted in providing physical comfort and teaching better strategies for patient mobility. The question that arose was: How do these professionals perceive the influence of their actions on the decision regarding the place of death for PAC? Thus, the fifth and final focus group consisted of 22 professionals, including 17 nurses, two nursing technicians, and three physical therapists. Chart 1 presents, for illustrative purposes, examples of questions used with members of the five sample groups, which were adjusted according to the ongoing comparativ

**Chart 1:** Examples of additional questions used in the five sample groups – PR, Brazil, 2025.

Group	Composition	Additional questions for each group
1	Family members of people who have died from cancer	Where did your family member pass away (at home, in the hospital, etc.)? How did it happen? Did you discuss where it would be best for this to happen? Who was involved in that discussion? Now, sometime later, what is your opinion on that decision? Why? Please tell us about it.
2	People with advanced cancer	Have you ever thought about or talked about where you would like to be cared for at the end of your life? Who have you talked to about this? What is your opinion on choosing the place of death for a family member? Who should decide this? Why? Tell us more about this.
3	Physicians	In your experience, how do you perceive that palliative care patients and their families react to the choice of place of death? Is a decision made? If so, who participates in it? What factors influence these decisions in clinical practice? Tell me more about the decisions regarding the place of death for palliative care patients you currently care for or have cared for.
4	Psychologists and social workers	Tell me about the place of death for palliative care patients you are currently caring for or have cared for in the past. Have you ever been asked to provide clarification or assist in the decision regarding the place of death? How do you perceive your role with these individuals and their families regarding the decision about the place of death? Please elaborate on this.
5	Nurses, nursing assistants, and physical therapists	The same questions presented to members of Group 4, but focused on the context of direct care.

### Data Collection

Data were collected through open-ended interviews, which were audio-recorded with participants’ consent, conducted between February 2023 and November 2024, either in person (30 participants) or via video call (66 participants). The interviews lasted between 20 and 90 minutes and were all conducted by the principal investigator, a nurse with experience in caring for people with cancer and conducting qualitative interviews, who had no prior relationship with the stud

Throughout the data collection and analysis process, the researcher employed reflexivity and theoretical sensitivity by drafting analytical and reflective memos after the interviews and during the coding stages. These records made it possible to articulate, monitor, and keep in check preconceptions and professional experiences related to palliative and end-of-life care. More specifically, the researchers had an empirical perception that dying at home would facilitate a “good enough” death. However, the data also indicated that the hospital setting could be the preferred place of death for some PACs and their families. In this sense, the researchers realized that their preconceptions could not take precedence over what the data revealed, thus avoiding their uncritical incorporation into the analysis and allowing categories to emerge from the findings, in accordance with the method of constan^(13)^

### Data Processing, Analysis, and Theoretical Validation

The interviews were transcribed in full without the use of *software* by the same researcher and preferably on the same day they were conducted, which allowed the transcripts to be enriched with recollections of nonverbal behaviors. Data analysis followed the stages of initial and focused coding, with the support of the Max QDA^®^ software, which was used as an auxiliary tool in organizing the data, without interfering in the analytical process. In the initial coding, the principal investigator segmented the data into smaller units, analyzing them word by word, line by line, and incident by incident, allowing for a detailed exploration of the participants’ meanings and experiences. This procedure enabled the identification of patterns and variations, fostering a deeper understanding of the phenomenon under investigation.

The entire analytical process was initially conducted by the principal investigator, who met monthly with the group’s senior researcher to present and discuss the findings. At that time, differences of opinion were explored until consensus wa

In focused coding, the concepts were progressively refined and gained greater analytical prominence, resulting in the emergence of subcategories and categories, and in the identification of the central category, which holds the greatest explanatory power and organizes the theoretical structure of the analysis: the decision-making process regarding the place of death in the oncological context. During this process, it was possible to identify theoretical saturation, characterized by the absence of findings that would allow for abstraction and advancement in theorization through the identification of new theoretical insights or new properties for the central theoretica^(13)^

Memos and analytical diagrams supported the construction of the substantive theory, which underwent a two-stage validation process: a return to the raw data and evaluation by ten individuals, including two PAC members, two relatives of people who died of cancer, and six professionals specializing in palliative care (a psychologist, a physical therapist, three nurses, and a palliative care physician). On this occasion, it was determined that no substantial changes were necessary to the central category, given that the new interviewees recognized themselves in the theoretical model presented.

### Ethical Considerations

The study complied with national and international guidelines for research involving human subjects, including the Guidelines for Research Procedures at Any Stage in a Virtual Environment, and was approved by the Human Research Ethics Committee of the signatory institution (CAAE 71635923.8.0000.0104; Opinion No. 6.224.766).

All participants were informed in advance about the purpose of the research and gave their consent to participate by signing the Informed Consent Form, either in person or virtually. To ensure anonymity in the presentation of the results, excerpts from the testimonies are identified with the letters FA, PAC, MED, PS, AS, ENF, TE, and FISIO to designate, respectively: family member, person with advanced cancer, physician, psychologist, social worker, nurse, nursing technician, and physical therapist, followed by a number indicating the order in which the interview was conducted within the respectiv

## RESULTS

The 96 study participants were from nine Brazilian states located in four regions (South, Southeast, Midwest, and Northeast). The group was predominantly female (75 women/21 men), with ages ranging from 22 to 82 years. The participants included PACs, relatives of people who died of cancer, and healthcare professionals from various fields, all residing in urban areas. This diversity of profiles allowed for multiple perspectives on the decision-making process regarding the place of death (Chart 2

**Chart 2:** Characterization of participants by sample groups – PR, Brazil, 2025.

Sampling group	N	Gender (Female/Male)	Age groups	Profile
GA1 – Relatives of deceased individuals	15	10F / 5M	22 - 70 years old	Six children, five spouses, and others with close ties (mother, daughter-in-law, niece, granddaughter). Nine deaths occurred in the hospital, one at an emergency care unit (UPA), and five at home. Seven people had health insurance.
GA2 – People with advanced cancer	24	23F / 1M	25 - 64 years old	15 lived with a partner; nine were receiving treatment through the SUS (Brazilian Unified Health System) and 14 through private or supplemental care. Four PACs were monitored by multidisciplinary teams specializing in palliative care; eight had a ADLW.
GA3 – Physicians	20	12F / 8M	30 - 58 years old	11 oncologists (two surgeons), nine palliative care specialists (four in home care programs). Five of them worked in charitable hospitals, seven in public hospitals, and two in private hospitals. Five provide home PC.
GA4 – Psychologists and Social Workers	15	13F / 2M	25 - 72 years old	11 psychologists and four social workers. Four of them worked in hospitals, one in a hospice, four in home care services, five in private practices, and two in a nonprofit organization for people with cancer.
GA5 – Nurses, Nursing Technicians, and Physical Therapists	22	17F / 5M	26 - 55 years old	17 nurses, two nursing technicians, and three physical therapists. Of these, 14 worked in the SUS in hospital settings (outpatient clinics, inpatient wards, ICUs, and PC), five in UCC affiliated with private health plans, and one in a private palliative care service.
Total	96	75F / 21M	22 - 82 years old	A variety of family and professional roles in different care settings.

Note: F = female; M = male; PC = palliative care; SAMU = Mobile Emergency Care Service; DAV = Advanced Directive/Living Will; UPA = Emergency Care Unit; UCC = Continuous Care Units; SUS = Unified Health System.

Geographic and institutional diversity allowed for the identification of four care scenarios: Scenario I (SUS with a palliative care team): multidisciplinary care with occasional home care; Scenario II (SUS without a structured palliative care program): care primarily provided by oncologists, with support from the Family Health Strategy or the “Melhor em Casa” (Better at Home) Program; Scenario III (private health plan with its own network and structured PC): specialized team, 24-hour home care, and medication provision; Scenario IV (private health plan without a PC structure or private care): oncologist-centered care, with patients seeking complementary suppor

Analysis of data from the five SGs studies enabled the development of a substantive theory titled “The social construction of the place of death in advanced cancer: between mediations and obstacles,” comprising two categories: 1) Enabling choices and mediating meanings: communication as a bridge between desires and realities, and 2) Systemic barriers: obstacles to the construction of shared meanings. This article will present the first category and its subcategories, namely: 1) The place of death as a symbolic space and 2) Relational dynamics: conflict, mediation, an

**Figure 1:**
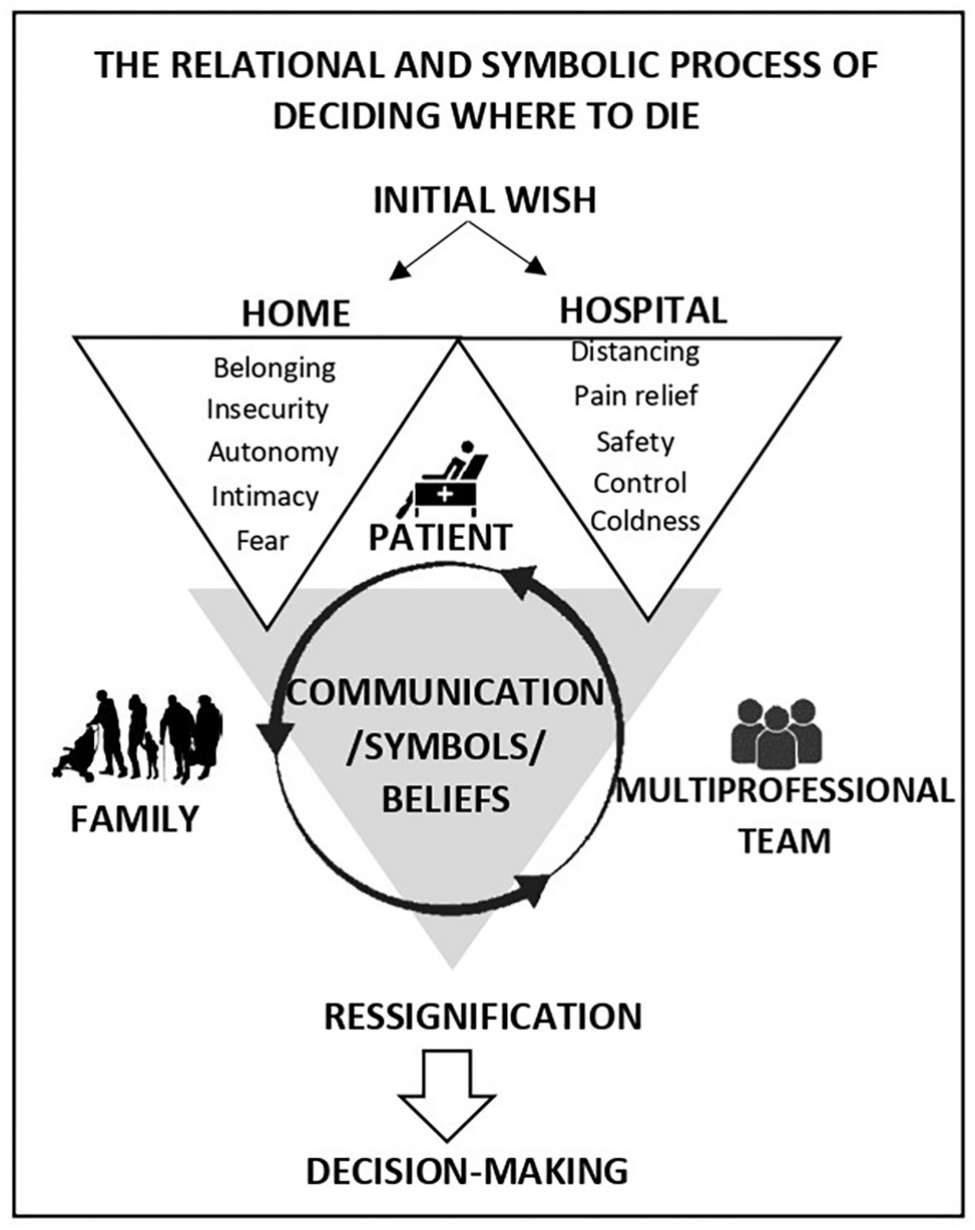
Diagram summarizing the processes experienced/identified in the category – Maringá, PR, Brazil, 2025.

Figure 1 illustrates the contrast between hospital and home as “choice” options for the place of death. It is evident that there is no unanimity in the choice; in reality, for many, the hospital represents both a sense of security, control, and pain relief as well as coldness and emotional detachment; whereas the home conveys, at certain moments, an idea of respect for autonomy, intimacy, and a sense of belonging, but at other times is perceived as a source of fear and insecurity. Decision-making involves a process of negotiation and consensus shaped by the consideration of subjective factors (beliefs, spirituality, memories), institutional factors (protocols, a culture of silence), and systemic factors (lack of resources, clinical conditions, caregiver burnout, communication breakdowns). Circular arrows highlight the importance of dialogue between PAC, family members, and healthcare professionals to mediate and facilitate choices, which, even if appropriate in one situation, may not be so i

### Enabling Choices and Mediating Meanings: Communication as a Bridge Between Desires and Realities

In this category, it is observed that communication plays a central role in mediating meanings, being constructed from symbolic interactions between PACs, family members, and healthcare professionals. This mediation enables the construction of shared meanings regarding the place of death, with the aim of respecting autonomy and preserving dignity until the end of life. In this context, it was identified that the appropriate management of pain and suffering constitutes the central symbolic element in the modulation, reframing, and definition of a new decision, which can either reaffirm the first decision or modif

A summary of the statements and subcategories that comprise the category and illustrate the findings is presented in Chart 3

**Chart 3:** Category, subcategories, and participants’ statements – PR, Brazil, 2025.

Enabling choices and mediating meanings: communication as a bridge between desires and realities	
*The place of death as a symbolic space*	*[...] I might change my mind, but for now, I’d prefer it to happen at the hospital, with proper care, partly to spare them the strain [...] because I have young children [...]. So, at the hospital, there are doctors and a staff. I feel safer at the hospital*. (PAC01, 41 years old) *Because at the hospital, they have resources we don’t have. So when he was sedated, he was calm, quiet, and then he passed away in the early morning*. (F09, 62 years old, wife) *[...] often the patient and the family feel safer at the hospital [...] having resources closer at hand, such as oxygen and immediate medication. Often there is a more emotionally vulnerable context [...] whether because family members would rather not witness the death or because there are children at home, so they choose to have the patient come to the hospital*. (MED-15, 34 years old) *I want to be at home with the people I love, in the place where I feel good, where I feel welcomed. This is my request*.(PAC15, 39 years old) *They decided to stay at home, close to family, for the comfort of home and convenience. There wasn’t much else to do*. (FA06, 33 years old, son) *The hospital environment isn’t as cozy as being at home with your closest family members. Being there in your own comfort, in your own little corner*. (PS08, 35 years old) *What’s important is being pain-free and in a more suitable environment*. (PAC03, 45 years old) *I think about being well cared for at the end of life, because pain isn’t pleasant*. (PAC23, 48 years old)
*Relational Dynamics: Conflict, Mediation, and Facilitation*	*My children and I made the decision. They said, “Dad, you can’t leave her here; we have to take her.” If she dies there, at least she’ll be receiving medical care*. (FA02, 69 years old, husband) *Her respiratory failure started to get much worse; she was practically in agony. Her husband called their three children and said: “We had promised we wouldn’t let her die in the hospital, that she would stay here at home with us, but seeing what she’s going through now, I can’t let her suffer like this. If they put at least a nasal catheter in her at the hospital so she can breathe until she passes away, that’s better.” So they decided to take her; it was a discussion among them*. (FA05, 22 years old, niece). *There are situations where the patient wants to go home, but the family doesn’t. To care for them, you need a rotation; you have to invest in professionals. So the family says: ‘There’s no way,’ you’re staying here in the hospital*. (ENF17, 26 years old) *If we took her to the hospital, maybe someone would stay with her, or she’d end up in an ICU and die there alone. She didn’t want to go either, so we decided to go through this ordeal, because it was an ordeal. My sister couldn’t handle it... she even called the ambulance. I’m a little calmer and stayed by her side the whole time. I was holding her hand when she died. The ambulance doctor said: “It’s up to you; we’re here to do whatever you want, but… the only difference is that here she’s surrounded by love, and at the hospital she’ll just be there… but if you want, we’ll take her.” So we decided not to take her.”* (FA11, 44 years old, daughter)

### The Place of Death as a Symbolic Space

The choice of a hospital is related to technical safety, as it is seen as a space that offers effective control of pain and suffering due to the immediate availability of resources and professional support. The choice of a hospital is also influenced by cultural values, religious beliefs, personal experiences with health care services, and experiences related to the end of life. For example, concerns about mitigating the suffering of family members—especially children witnessing the final moments—the increased burden on families during end-of-life care, and even the desire to avoid negative memories in the home environment all contribute to making the hospital the place of choice fo

On the other hand, the home is symbolized as a place of dignity and comfort and represents emotional connection, welcomingness, intimacy, respect for the autonomy of the terminally ill patient, and the strengthening of family bonds. The pursuit of dignity and autonomy is intrinsically related to the meaning attributed to pain and suffering, with the effective management of these factors being the primary determinant in decision-making. When pain and suffering cannot be adequately controlled, the home ceases to be a viable option, highlighting the distance from the desired comfort.

Another relevant aspect is that adequate pain management symbolizes dignity and respect in the final moments of life. This management transcends physical control, reaching emotional and symbolic dimensions, which directly influences the perception of dignity and the feasibility of the chosen location for death to occur. When the control of pain and suffering is inadequate, the perception of dignity is compromised, and the hospital is reframed as a space of relief an

### Relational Dynamics: Conflict, Mediation, and Enabling

The pursuit of relief from or the absence of pain and suffering triggers a negotiation process regarding the place of death, involving people with cancer, their family members, and healthcare professionals. This dynamic, marked by conflicts, negotiations, mediations, and enabling, is permeated by communication, which acts as a central element to align expectations and facilitate decision-making.

Family dynamics illustrate the collective *Self*, in which meanings are negotiated among members. It is also noted that decision-making conflicts may arise from divergences between the wishes of the PAC and those of their family members. In general, conflicts emerge from different interpretations of what is “best” for the PAC. These conflicts are moments of symbolic negotiation, with meanings being adjusted or reaffirmed through mediation. The mediating agents in this process of continuous reframing can be family members or healthcar

Family members’ *Self* is also shaped through interaction, as they assume roles of caregiving, negotiation, and decision- making. Conflict among family members can be interpreted as a symbolic struggle to preserve the collective *Self* of the family. In these cases, healthcare professionals play a crucial role in facilitating this mediation; they are essential in reframing fears and expectations by enabling new experiences that can redefine the place of death and thus helping family members align their meanings and expectations. This mediation not only resolves conflicts but also creates shared meanings regarding the place o

## DISCUSSION

The results highlight the importance of professional mediation, active listening, and effective communication in facilitating the negotiation of shared meanings and decisions among patients, family members, and healthcare teams—aspects identified as fundamental for promoting person-centered care and quality of life at the end of life. Previous studies highlight that assertive communication and the involvement of nursing professionals in the decision-making process are crucial strategies for ensuring patient autonomy and strengthening the therapeutic relationship in the context of palliative care, contributing to more humanized practices aligned with patients’ individual values^(19,20)^.

The category “Enabling choices and mediating meanings: communication as a bridge between desires and realities” shows that the decision regarding the place of death constitutes a symbolic, interactional, and relational process. This study broadens our understanding of the phenomenon by highlighting that symbols such as hospital, home, pain, and dignity are integral to the meanings attributed to the place of death, which are constantly negotiated and made possible or impossible given the available structural and relational conditions. This knowledge provides theoretical foundations for palliative care practices and policies, a point reinforced by research demonstrating that symbolic elements guide both family members and multidisciplinary teams in the co-construction of these meanings^(21,22)^.

The meanings attributed to the place of death emerge from social relationships and are shaped by how individuals interpret their experiences^(12)^. Thus, the hospital is symbolized as a space of protection and technical care or, conversely, as a place of alienation and a breach of dignity, as revealed in this and other studies^(3,6)^. Consequently, the place of death acquires diverse meanings depending on the symbolic repertoire that the patient and their caregivers construct throughout the course of th

In non-oncological contexts, similar results have also been identified. International studies^(6,8,23)^ indicate that, in diseases such as dementia or chronic kidney disease, the hospital tends to be the predominant place of death, yet this is at odds with previously expressed preferences, primarily due to insufficient home care and family overload. This reinforces that the social construction of the place of death—sometimes as a space of dignity and connection, sometimes as a setting of rupture and institutionalization—is not exclusive to cancer patients but cuts across different illnes

Studies in Brazil^(7,24)^ indicate the predominance of the hospital as the place of death for people with cancer, especially in the face of clinical deterioration or a lack of home care^(7)^. In the present study, it also signified family protection, especially to prevent children from witnessing the terminal phase, thereby preventing emotional impacts and alleviating the burden on loved ones. On the other hand, the home symbolized respect for dignity and autonomy, as well as the maintenance of emotional connection, provided by proximity to loved ones^(6)^ and the preservation of the *Self*. However, when family members do not feel prepared to face the natural progression of end-of-life care and begin to experience great suffering, this preference may be reinterpreted, making the hospital an inevitable choice^(5)^. Thus, dying at home represents not merely a choice of location but an affirmation of the *self* within its emotional environment, demonstrating that the geography of care is, rather, a narrative o

However, it is necessary to consider that regional differences in the availability of specialized services and difficulties in access can influence the choice of place of death. Furthermore, according to some reports, in the context of cancer, there are striking differences in access to and availability of services, even within the same region or state, depending on the place of residence—whether in the capital or the interior, or in an urban or rural area. This fact certainly has important implications for equity in palliative care. A study on the temporal trend of mortality from neoplasms in Brazil between 2002 and 2022 found, for example, that São Paulo and Rio Grande do Sul had high rates of deaths in the hospital setting, possibly associated with a greater supply of specialized oncology services. On the other hand, states in the North and Northeast, such as Maranhão and Piauí, recorded increasing trends in home deaths, which may be related to low coverage of specialize^(24)^

Despite these issues, evidence indicates that robust public policies, which guarantee access, attentive care, and continuous support, increase the likelihood of aligning the place of death with the patient’s preferences, promoting dignity, comfort, and a reduction in unnecessary interventions at the end of life^(25,26)^. Thus, the PNCP established in Brazil in 2024^(10)^ represents an important step toward overcoming structural barriers, expanding access to home care, strengthening multidisciplinary teams, and aligning with successful international models^(26)^. To this end, Primary Health Care must be restructured to include palliative care teams so that patients and their families can rely on support for managing pain and suffering, thereby avoiding the need to seek hospital care when suffering become^(27)^

The PNCP aims to develop initiatives that promote palliative care education, encouraging the training and continuing education of professionals within the Health Care Network^(28)^, in which the nursing team plays a pivotal role in integrating other team members with the patient and family car

Additionally, the construction of the self in the terminal care process, understood as the way a person sees themselves and is recognized by others^(29)^, is constantly mobilized. The desire to remain at home expresses the intention to maintain the continuity of the self in the face of finitude; whereas hospitalization, even though it restricts control, can symbolize protection and hope for support in the face of suffering. This reiterates that preferences fluctuate according to the negotiation between personal values and clinical needs. Professional mediation emerges as a key element in the reconstruction of meanings. Professionals who listen and welcome expand possibilities by acting as symbolic mediators of the dying process^(29)^. By assuming the role of the other^(30)^, these professionals facilitate the expression of the self and contribute to a shared decision grounded in real values and desires. Thus, family conflicts emerged as a factor in the decision-making process regarding the place of death and highlight the importance of the nurse’s role not only as a clinical caregiver but also as a communicational mediator among famil

The palliative care literature emphasizes that nursing professionals can implement family mediation techniques through strategies such as structured family meetings, the use of empathetic and facilitative communication, active listening, and guidance for resolving differences in values and expectations, thereby promoting shared understanding and joint decisions regarding care^(31)^. These approaches can help reduce tensions, strengthen emotional support, and align choices regarding the place of death with the preferences of patients and their families, integrating clinical and communication skills into holisti

Furthermore, it is necessary to recognize the limitations posed by intractable family conflicts, precarious structures, and institutional resistance to active listening, which constitute ethical and contextual constraints that strain this mediation and impact the feasibility of the desired place of death. This is the moment when a family conference should be held, as a therapeutic tool to resolve conflicts and ethica^(32)^

In this context, the family conference is a therapeutic intervention, mediated by professionals from the palliative care team, constituting a structured space for communication that fosters active listening, the expression of values, and the negotiation of expectations among patients, family members, and the healthcare team, thereby reducing conflicts and promoting shared decisions. By acting as a communication mediator, the professional - often the nurse - helps align care choices, including the place of death, with the patient’s preferences, strengthening mutual understanding and person-centere^(32)^

Interaction between patients, family members, and healthcare professionals can trigger symbolic conflicts that require effective communication and consensus-building. In contexts such as China, the “silence barrier”, concealing the condition for emotional protection, limits autonomy and hinders shared decision-making^(33)^, and similar scenarios are replicated in Brazil, exacerbated by the fragmentation of services and the absence of integrate^(7)^

Furthermore, healthcare institutions face challenges in communicating about dying and death, as biomedical models still predominate and technical language often obscures the listening to and expression of the PAC’s wishes^(9,34)^. In this environment, the “silence barrier,” though culturally driven, hinders the patient’s active participation in the decision-making process^(30,33)^, disrupting the shared construction of meaning and preventing the *self* from expressing itself in the face o

Institutional interaction reduces death to a technical event, denying its symbolic, affective, and relational dimensions. Furthermore, it not only obscures or distorts the meanings attributed by the PAC and their family members but also contributes to the absence of adequate advance planning for the final moments of life. Therefore, clear and empathetic communication among professionals, patients, and family members is fundamental to mitigating barriers and facilitating decisions^(5,8)^. However, professional mediation, though desirable and powerful, depends on structure, time, emotional preparedness, and institutional legitimacy.

Faced with this communicational challenge, nurses, as symbolic mediators and key professionals in daily care, must enhance their skills in therapeutic listening and confident communication. To this end, they must know how to identify the appropriate moment to address questions about the dying process and death with the patient and their family members^(35)^, in order to reestablish horizontal interaction and the co-construction of meaning in the decision-makin

Resolution No. 564/2017 of the Code of Ethics for Nursing Professionals highlights the nurse’s responsibility to promote conditions for patient autonomy in decision-making regarding care and comfort^(36)^. Internationally, the Code of Ethics of the International Council of Nurses^(37)^ also reinforces respect for human dignity, patients’ rights, and their choices at all stages of life, including end-of-lif

These national and international ethical guidelines demonstrate that nursing practice must be guided by a humanized approach, sensitive to the uniqueness of each person and promoting autonomy, regardless of the setting^(38)^. In this context, the nursing team acts as a symbolic mediator, since the choice of place of death requires not only infrastructure but also horizontal relationships, active listening, and symbolically welcoming environments, enabling personalized actions^(20)^ that address specific needs and value the “today” with its limitations and possibilities, enhancing the quality of the remainin

The recognition that the choice of place of death is a symbolic construction requires that, in addition to infrastructure, people be assured the power to attribute meaning to this decision through horizontal interactions and welcoming environments^(5)^. Care models that disregard language, values, and social dynamics tend to reproduce inequalities and dehumanizing practices, whereas a meaning-centered approach can strengthen clinical effectiveness, social justice, and ethical care at the end of life. Therefore, recognizing the relational and symbolic nature of these decisions guides practices and policies that value patient agency, promote equity, and enable truly humanized care in palliativ

Among the study’s potential limitations are the retrospective interviews with family members, which may involve subjective reconstructions of final events. Furthermore, the predominance of remote interviews—which, on the one hand, expanded the geographic reach—may have limited the perception of nonverbal cues. Finally, the concentration of participants from urban areas and of female gender did not allow for rural realities and different gender perspectives to b

Thus, considering that the meanings attributed to the place of death are negotiated and made feasible or infeasible in light of different structural and relational conditions, it is believed that an appreciation of the nuances of urban and rural contexts and the participation of a larger number of male individuals, especially among PACs and family members, can enrich our understanding of the phenomenon. We therefore point to the need for future research in diverse car

## CONCLUSION

The substantive theory constructed describes the decision regarding the place of death as a symbolic, interactional, and dynamic process. The analysis of the narratives revealed layers of negotiated meanings surrounding the hospital and the home, influenced by cultural values, prior experiences, institutional conditions, and available resources. This approach made it possible to understand how these meanings are constructed, re-signified, and shared, allowing for the identification of factors that shape this decision in the oncological context. The choice of place of death is an expression of the *self* mediated by language, active listening, and the ability to assume the role of the other. The interdependence between the patient, family members, and the multidisciplinary team—especially the role of the nurse as a symbolic mediator—is fundamental to ensuring time, Welcoming, and dignity. Implications for practice and public policy include investment in home care, supported by the PNCP. Future longitudinal studies should compare realities before and after the implementation of the PNCP to assess its impact on enabling choices, mediating meanings, and overcoming systemi

## Data Availability

The entire dataset supporting the results of this study is available upon request to the corresponding author.
