# Supplementary material for: Relational and symbolic process: decisions regarding the place of death in the context of advanced cancer
Source: Rev Esc Enferm USP. 2026 Jul 31;60:e20250405. doi: 10.1590/1980-220X-REEUSP-2025-0405en (PMC13432645; doi:10.1590/1980-220X-REEUSP-2025-0405en)
Supplement: Translation

# Translation: Processo relacional e simbólico: decisão sobre o local de morte no contexto do câncer avançado*

**DOI:** 10.1590/1980-220X-REEUSP-2025-0405pt

**Authors:** Patrícia Chatalov Ferreira, Marcelle Miranda da Silva, Mayckel da Silva Barreto, Esperanza Begoña Garcia-Navarro, Cristina Garcia-Vivar, Eleandro do Prado, Rudval Souza da Silva, Sonia Silva Marcon

**Affiliations:** 1 Universidade Estadual de Maringá, Programa de Pós-Graduação em Enfermagem, Maringá, PR, Brasil.; 2 Universidade Federal do Rio de Janeiro, Programa de Pós-Graduação em Enfermagem, Rio de Janeiro, RJ, Brasil.; 3 Universidade de Huelva, Departamento de Enfermagem, Huelva, España.; 4 Universidad Pública de Navarra, Departamento de Enfermagem, Pamplona, Navarra, España.; 5 Universidade Positivo, Colegiado de Enfermagem, Curitiba, PR, Brasil.; 6 Universidade do Estado da Bahia, Programa de Pós-Graduação em Ciências do Cuidar em Saúde, Senhor do Bonfim, BA, Brasil.

**Keywords:** Cuidados Paliativos, Neoplasias, Tomada de Decisão, Família, Enfermagem, Teoria Fundamentada.

## Abstract

**Objetivo:**

Construir uma teoria substantiva acerca dos significados atribuídos e os fatores que influenciam a decisão sobre o local de morte no contexto do câncer avançado.

**Metodologia:**

Utilizados como referenciais o Interacionismo Simbólico e a Teoria Fundamentada nos Dados. Foram realizadas entrevistas áudio-gravadas, presenciais ou remotas, entre 2023 e 2024, com 96 participantes de nove estados localizados em quatro regiões brasileiras, que integraram cinco grupos amostrais.

**Resultados:**

O hospital significa espaço de proteção, alívio da dor e do sofrimento/sobrecarga familiar e o domicílio um ambiente de promoção da dignidade e autonomia do doente e conexão afetiva com familiares, acompanhado de insegurança quanto ao manejo da dor e sofrimento. A mediação profissional e a escuta ativa favorecem a negociação de significados, enquanto barreiras estruturais e comunicacionais limitam a viabilização das escolhas.

**Considerações Finais:**

A decisão sobre o local de morte constitui processo simbólico, relacional e dinâmico, moldado por conflitos familiares, limitações na estrutura física e interações entre pacientes, familiares e equipe multiprofissional. É importante a efetivação das políticas públicas que possibilitem escolhas coerentes com os valores da pessoa com doença terminal.

## INTRODUÇÃO

A decisão sobre o local de morte no contexto do paciente com câncer avançado tem se tornado uma questão central, especialmente diante do aumento progressivo das neoplasias e condições crônicas complexas, o que intensifica desafios para os sistemas de saúde em países em desenvolvimento, como o Brasil^(1)^. A pandemia de COVID-19 agravou essa realidade, postergando diagnósticos e comprometendo o acesso aos serviços, o que contribuiu para a identificação tardia de casos em estágio avançado^(2)^.

O enfrentamento de uma condição irreversível, embora não implique em morte imediata, exige decisões complexas relacionadas aos cuidados no fim da vida. Entre elas, destacam-se a definição do ambiente em que se deseja que ocorra a morte, a escolha do local para os cuidados paliativos e a organização do cuidado^(3)^. A noção pragmática de “morte suficientemente boa” (*good enough death*), concentra-se na oferta de um cuidado adequado e no controle dos sintomas físicos, emocionas e espirituais. Tal deslocamento explicaria a divergência entre as expectativas dificilmente alcançáveis de uma proposta de morte idealizada e a realidade cotidiana do trabalho nos serviços, marcada por complexidade e heterogeneidade^(4)^. Desse modo, as decisões sobre o local de ocorrência da morte, são atravessadas por fatores clínicos, emocionais, sociais e simbólicos, o que explica a diversidade de preferências e motivações que orientam as escolhas^(5)^. Assim, compreender esse processo decisório requer considerar não apenas aspectos objetivos, mas também os significados construídos nas interações sociais.

Grande parte dos estudos que abordam a escolha do local de morte foi conduzida em países desenvolvidos, apresentando enfoques restritos que pouco consideram a natureza interativa do processo decisório^(3,5,6)^. Essa lacuna torna-se ainda mais relevante quando observada no contexto brasileiro, marcado por desigualdades de acesso aos serviços de saúde e diversidade cultural, fatores que tornam esse debate ainda mais complexo^(3,7)^.

Na literatura nacional e internacional, os estudos quantitativos relacionam a escolha do local de morte a características sociodemográficas, como idade, estado civil e suporte familiar^(3,5,6,7,8)^. Contudo, permanecem escassas as investigações qualitativas que explorem de forma aprofundada os significados atribuídos e as interações sociais que sustentam esse processo^(9)^. Essa carência de estudos não apenas dificulta a compreensão mais ampla e contextualizada do fenômeno, como também limita a produção de marcos teóricos e práticos capazes de orientar políticas e práticas profissionais mais sensíveis à realidade vivida pelas pessoas. No Brasil, tal escassez é ainda mais evidente em periódicos da área de enfermagem, o que reforça a necessidade de estudos que ampliem a compreensão desse fenômeno sob uma perspectiva interacional e prática^(7)^.

No contexto nacional, as investigações sobre o processo decisório acerca do local de morte ainda permanecem incipientes e fragmentadas, sobretudo quando se considera a complexidade relacional que envolve o tripé pacientes, familiares e equipes de saúde. A originalidade deste estudo reside na abordagem compreensiva e interacional adotada, que privilegia a construção de significados compartilhados e as dinâmicas comunicacionais implicadas nesse processo. Ao propor uma teoria substantiva ancorada em evidências empíricas, o trabalho amplia o entendimento do fenômeno para além das variáveis individuais, oferecendo uma leitura inovadora que integra dimensões subjetivas, sociais e institucionais dos cuidados no fim da vida^(7)^.

Além disso, cabe realçar que a presente investigação também se sustenta na consonância com as diretrizes da Política Nacional de Cuidados Paliativos (PNCP)^(10)^, a qual enfatiza a centralidade da pessoa e da família nas decisões sobre o cuidado, bem como a necessidade de ampliar o acesso equitativo a serviços de qualidade em todos os níveis de atenção, especialmente num país como o Brasil no qual o ranqueamento sobre a qualidade dos cuidados paliativos o coloca na posição 79 num rol de 81 países^(11)^. Compreender os fatores e significados que orientam a decisão sobre o local de morte contribui diretamente para a implementação dessa política, ao subsidiar práticas assistenciais mais humanizadas, comunicacionais e culturalmente sensíveis. Além disso, pode-se pressupor que os achados venham a apoiar gestores e profissionais na formulação de estratégias voltadas à organização das redes de atenção, fortalecendo os cuidados paliativos como componente essencial do sistema de saúde e reafirmando o papel da enfermagem na coordenação e na integralidade dos cuidados no fim da vida.

Diante dessa lacuna, o objetivo do estudo foi construir uma teoria substantiva acerca dos significados atribuídos e os fatores que influenciam a decisão sobre o local de morte no contexto do câncer avançado. Tal compreensão possui implicações diretas para a prática da enfermagem oncológica e para a organização de serviços de cuidados paliativos, ao favorecer estratégias de cuidado mais sensíveis às necessidades de pessoa com câncer avançado e seus familiares.

## MÉTODO

### Desenho do Estudo e Referencial Teórico e Metodológico

Trata-se de uma pesquisa qualitativa, de caráter explicativo, que utilizou como referencial teórico o Interacionismo Simbólico (IS), segundo o qual, os significados atribuídos às situações ou coisas emergem das interações sociais e das experiências vividas^(12)^. A Teoria Fundamentada nos Dados (TFD), vertente construtivista, foi a opção metodológica. Esta vertente pressupõe que as experiências do pesquisador tanto, quanto dos participantes, são relevantes e influenciam a interpretação dos dados e, por conseguinte, na construção do fenômeno^(13)^. Na elaboração do relatório de pesquisa foram seguidas as diretrizes *Consolidated Criteria for Reporting Qualitative Research* (COREQ).

### Local, População e Critérios de Seleção

Participaram do estudo 96 pessoas, distribuídas em cinco grupos amostrais (GA) constituídos por familiares de pessoas falecidas por câncer há no máximo um ano, pessoas com câncer avançado, médicos, psicólogos e assistentes sociais e por fim enfermeiros e fisioterapeutas.

Os critérios de inclusão para pacientes e familiares foram ter 18 anos ou mais e capacidade de comunicação verbal. Não foram incluídos indivíduos com instabilidade emocional ou sem condições de acesso a recursos tecnológicos, no caso das entrevistas remotas. Já para os profissionais, considerou-se ter pelo menos seis meses de experiência na assistência a pessoas com câncer.

### Amostragem Teórica

A coleta de dados teve início com quatro familiares de pessoas falecidas em decorrência do câncer, indicadas por profissionais de uma instituição filantrópica atuante no município sede do estudo. Ao final das entrevistas foi solicitada a indicação de novos potenciais participantes, tal como proposto pela técnica de amostragem não probabilística denominada *Snowball Sampling* (bola de neve)^(14)^. Entretanto, houve dificuldades para efetivar a participação dos indicados e, considerando que as redes sociais têm sido utilizadas de maneira satisfatória como cenário de estudos qualitativos, inclusive na abordagem de temas sensíveis como a violência doméstica^(15)^, optou-se por explorar outros contextos culturais e socioeconômicos, recrutando familiares e pessoas com câncer avançado que compartilhavam suas vivências usando-se como recursos as redes sociais.

Estudos incluídos em uma revisão^(16)^ indicam que o recrutamento de participantes por meio de redes sociais é uma estratégia eficaz e de baixo custo em pesquisas em saúde, desde que acompanhado de critérios rigorosos de verificação de credibilidade, como triagem prévia, cruzamento de informações e mecanismos para identificação de perfis inadequados. Assim, em busca por mitigar riscos éticos, a seleção desses participantes foi conduzida de forma criteriosa, considerando além dos aspectos citados, a mediação por administradores de grupos e a confirmação da elegibilidade durante o contato inicial, conforme recomendações metodológicas para coleta remota de dados qualitativos^(17)^. Ademais, os riscos éticos inerentes a esse tipo de recrutamento foram mitigados por meio da obtenção de consentimento informado, garantia de confidencialidade, atenção à privacidade dos participantes e monitoramento contínuo de possíveis desconfortos emocionais, em consonância com a literatura especializada^(18)^.

O primeiro grupo amostral (GA) foi constituído por 15 familiares de pessoas falecidas em decorrência do câncer (cinco em entrevistas presenciais e 10 em entrevistas *online*). A análise dos dados desse grupo mostrou que a decisão sobre o local de morte é influenciada pelas dinâmicas familiares e pela comunicação com profissionais de saúde, marcada pelo Cerco do Silêncio e por um Modelo Paternalista. Surgiu assim a hipótese de que as estratégias de “proteção” adotadas pelos familiares, podem interferir na autonomia da pessoa com câncer avançado (PCA) em relação à decisão sobre o local de morte. Deste modo, definiu-se pela necessidade de incluir estas e ouvi-las em relação à autonomia na escolha do local de morte.

Assim, o segundo GA foi constituído por 24 PCA, identificadas em perfis no Instagram que mencionavam em suas biografias ou publicações públicas, termos como “câncer avançado”, “metástase”, “metastático”, “paliativo” e “paliativa”. Os convites para participação foram enviados em mensagens no *direct*, e quando aceito, era agendado dia e horário para a realização da entrevista por videochamada no *Google Meet.* A questão norteadora neste grupo foi “Conte-me a sua história com o câncer”. A abordagem sobre a opção pelo local de morte só ocorreu em momento oportuno, após a pesquisadora sentir abertura para isto, de modo a não desencadear gatilhos e sofrimento emocional.

Durante a coleta e análise das entrevistas constatou-se que a PCA demonstrava preferência sobre o local de morte, todavia foi percebido que haviam pessoas que não tinham espaço para falar sobre isto com seus familiares ou com profissionais de saúde, em especial com o seu médico. Esse contexto desencadeou o seguinte questionamento: Qual a opinião de médicos sobre a escolha do local de morte de PCA? A hipótese era de que os médicos acreditam que a PCA não tem condições de fazer esta escolha.

Desse modo, o terceiro GA foi constituído por 20 médicos. A questão norteadora foi: “Fale-me acerca das decisões sobre o local de morte de PCA que você acompanha ou acompanhou”. A análise dos dados deste grupo mostrou que oncologistas e paliativistas abordam de formas diversas questões sobre a morte, cuidados paliativos e local de morte com familiares e com a própria PCA. Surgiu assim o seguinte questionamento: Qual é o papel de outros profissionais de saúde no processo decisório? A hipótese era de que o suporte psicológico a pacientes e familiares influenciava a aceitação da terminalidade e a decisão sobre o local de morte.

Por conseguinte, o quarto GA foi constituído por 15 profissionais, sendo 11 psicólogos e quatro assistentes sociais. Observou-se que os primeiros auxiliam os médicos na comunicação difícil, trabalham diretamente com as dimensões emocionais e psicológicas acerca da terminalidade, enquanto os Assistentes Sociais se mostraram fundamentais no suporte aos familiares, lidando com questões logísticas, sociais e financeiras.

Mediante os dados obtidos nesse grupo, observou-se que, os fatores sociais e econômicos influenciam fortemente a decisão pelo local de morte e que o suporte logístico viabiliza o cuidado no fim de vida no domicílio. Para além, alguns relatos de PCA e de familiares destacaram que os enfermeiros se envolvem diretamente na prestação dos cuidados e no apoio emocional, tanto no hospital como no domicílio, além de frequentemente fazerem a mediação entre médicos e familiares/pacientes e que os fisioterapeutas auxiliavam na oferta do conforto físico e no ensinamento de melhores estratégias para mobilidade do paciente. O questionamento que surgiu foi: Como estes profissionais percebem a influência de sua atuação na decisão sobre o local de morte de PCA? Logo, o quinto e último GA foi composto por 22 profissionais, sendo 17 enfermeiros, dois técnicos de enfermagem e três fisioterapeutas. No Quadro 1 são apresentadas, de forma ilustrativa, exemplos de questões utilizadas junto aos integrantes dos cinco grupos amostrais, as quais eram ajustadas conforme a análise comparativa constante.

**Quadro 1:** Exemplos de perguntas adicionais usadas nos cinco grupos amostrais – PR, Brasil, 2025.

Grupo	Composição	Perguntas adicionais em cada grupo
1	Familiares de pessoas falecidas em decorrência do câncer	Onde o seu familiar faleceu (casa, hospital...). Como ocorreu? Vocês chegaram a discutir onde seria melhor que isto ocorresse? Quem participou desta discussão? Hoje, depois de passado um tempo, qual sua opinião sobre esta decisão? Por quê? Fale sobre isto.
2	Pessoas com câncer avançado	Em algum momento você já pensou ou conversou sobre onde (local) gostaria de ser cuidado(a) no fim da vida? Com quem já conversou? Qual sua opinião sobre a escolha do local de morte de um familiar? Quem deve decidir sobre isto? Por quê? Fale mais sobre isto.
3	Médicos	Na sua experiência, como você percebe que pacientes em cuidados paliativos e seus familiares reagem em relação à opção pelo local de morte? Ocorre uma decisão? Se sim, quem participa da mesma? Quais fatores influenciam essas decisões na prática assistencial? Fale-me mais acerca das decisões sobre o local de morte de PCA que você acompanha ou acompanhou.
4	Psicólogos e assistentes sociais	Fale-me acerca do local de morte de PCA que você acompanha ou acompanhou? Em algum momento você foi solicitado a prestar esclarecimentos ou ajudar na decisão sobre o local de morte? Como você percebe seu papel junto a estas pessoas e seus familiares em relação à decisão sobre o local de morte? Fale mais sobre isto.
5	Enfermeiros, técnicos de enfermagem e fisioterapeutas	Mesmas questões apresentadas aos integrantes do grupo 4, contudo focadas no contexto do cuidado direto.

### Coleta de Dados

Os dados foram coletados mediante entrevistas abertas, áudio gravadas após autorização, realizadas entre fevereiro de 2023 e novembro de 2024, presencialmente (30 pessoas) ou por videochamada (66 pessoas). As entrevistas tiveram duração entre 20 e 90 minutos e todas foram conduzidas pela pesquisadora principal, enfermeira com experiência em acompanhamento de pessoas com câncer e na realização de entrevistas qualitativas, a qual não tinha qualquer tipo de relação com os participantes do estudo.

Durante todo o processo de coleta e análise dos dados a pesquisadora empregou a reflexividade e sensibilidade teórica, mediante a elaboração de memos analíticos e reflexivos após as entrevistas e durante as etapas de codificação. Esses registros possibilitaram explicitar, monitorar e manter em suspensão as pré-compreensões e experiências profissionais relacionadas aos cuidados paliativos e no fim da vida. De maneira mais concreta exemplifica-se que os pesquisadores tinham uma percepção empírica de que a ocorrência da morte em casa favoreceria uma morte suficientemente boa. Entretanto, os dados também assinalavam que o ambiente hospitalar poderia constituir local de escolha para a morte por algumas PCA e suas famílias. Nesse sentido, as pesquisadores perceberam que suas preconcepções não poderiam predominar diante daquilo que os dados revelavam, evitando sua incorporação acrítica na análise e permitindo que as categorias emergissem a partir dos achados, conforme o método da comparação constante^(13)^.

### Tratamento, Análise dos Dados e Validação Teórica

As entrevistas foram transcritas na íntegra sem uso de *software*, pela mesma pesquisadora e preferencialmente, no mesmo dia de sua realização, o que permitiu enriquecer os registros com lembranças de comportamentos não verbais. A análise dos dados seguiu as etapas de codificação inicial e focalizada, com apoio do *software* Max QDA^®^ que foi utilizado como ferramenta auxiliar na organização dos dados, sem interferência no processo analítico. Na codificação inicial a pesquisadora principal segmentou os dados em unidades menores, sendo analisados palavra por palavra, linha por linha e incidente por incidente, permitindo uma exploração detalhada dos significados e experiências dos participantes. Esse procedimento possibilitou a identificação de padrões e variações, favorecendo uma compreensão aprofundada do fenômeno investigado.

Todo o processo analítico foi inicialmente realizado pela pesquisadora principal que mensalmente se reunia com a pesquisadora sênior do grupo, para apresentação e discussão dos achados. Nesse momento, divergências eram exploradas até a chegada do consenso.

Na codificação focalizada os conceitos foram progressivamente refinados e ganharam maior destaque analítico, resultando no surgimento de subcategorias e categorias e, na identificação da categoria central, que detém maior poder explicativo e organiza a estrutura teórica da análise: o processo de tomada de decisão sobre o local de morte no contexto oncológico. Durante esse processo foi possível identificar a saturação teórica, caracterizada pela ausência de achados que permitissem a abstração e avanço na teorização, mediante a identificação de novos *insights* teóricos, ou de novas propriedades para as categorias teóricas centrais^(13)^.

Memos e diagramas analíticos subsidiaram a construção da teoria substantiva, a qual passou por um processo de validação constituído de duas etapas: retorno aos dados brutos e avaliação por dez indivíduos, sendo duas PCA, dois familiares de pessoas falecidas por câncer e seis profissionais especializados em cuidados paliativos (uma psicóloga, um fisioterapeuta, três enfermeiros e um médico paliativista). Nesta ocasião foi identificado que não era necessária a realização de alterações consubstanciais na categoria central, tendo em vista que os novos entrevistados se reconheciam no modelo teórico apresentado.

### Aspectos Éticos

O estudo atendeu às prerrogativas nacionais e internacionais de pesquisa com seres humanos, respeitando inclusive as Orientações para Procedimentos em Pesquisas com Qualquer Etapa em Ambiente Virtual, sendo aprovado pelo Comitê de Ética em Pesquisa com Seres Humanos da instituição signatária (CAAE 71635923.8.0000.0104; Parecer nº 6.224.766).

Todos os participantes foram previamente esclarecidos sobre o objetivo da pesquisa e manifestaram anuência em participar, mediante a assinatura do Termo de Consentimento Livre e Esclarecido, de forma presencial ou virtual. Para assegurar o anonimato, na apresentação dos resultados, os extratos dos depoimentos estão identificados com as letras FA, PCA, MED, PS, AS, ENF, TE e FISIO para designar respectivamente: familiar, pessoa com câncer avançado, médico, psicólogo, assistente social, enfermeiro, técnico de enfermagem e fisioterapeuta, seguido de um número indicativo da ordem de realização da entrevista dentro do respectivo grupo.

## RESULTADOS

Os 96 participantes do estudo eram provenientes de nove estados brasileiros localizados em quatro regiões (Sul, Sudeste, Centro-Oeste e Nordeste). Houve predominância de pessoas do sexo feminino (75F/21M), com idades variando entre 22 e 82 anos. Os participantes foram PCA, familiares de pessoas que morreram por câncer e profissionais de diferentes categorias da área da saúde, todos residentes em área urbana. Essa diversidade de perfil possibilitou múltiplas perspectivas sobre o processo de decisão quanto ao local de ocorrência da morte (Quadro 2).

**Quadro 2:** Caracterização dos participantes segundo grupos amostrais – PR, Brasil, 2025.

Grupo Amostral	N	Sexo (F/M)	Idade (faixa etária)	Perfil
GA1 – Familiares de pessoas falecidas	15	10F / 5M	22 - 70 anos	Seis filhos(as), cinco cônjuges e os demais com vínculos próximos (mãe, nora, sobrinha, neta). Nove óbitos ocorreram no hospital, um em UPA e cinco no domicílio. Sete pessoas possuíam plano de saúde.
GA2 – Pessoas com câncer avançado	24	23F / 1M	25 - 64 anos	15 viviam com companheiro(a); nove faziam tratamento pelo SUS e 14 em serviço privado ou suplementar. Quatro PCA eram acompanhadas por equipes multidisciplinar especializadas em CP; oito tinham DAV declaradas.
GA3 – Médicos	20	12F / 8M	30 - 58 anos	11 Oncologistas (dois cirurgiões), nove paliativistas (quatro em programas domiciliares). Cinco deles atuavam em hospitais filantrópicos, sete em hospitais públicos e dois em privados. Cinco realizam CP domiciliares.
GA4 – Psicólogos e Assistentes Sociais	15	13F / 2M	25 - 72 anos	11 psicólogos e quatro assistentes sociais. Quatro deles atuavam em hospitais, um em hospice, quatro em serviço de atendimento domiciliar, cinco em consultórios e dois em instituição sem fins lucrativos para pessoas com câncer.
GA5 – Enfermeiros, Técnicos de Enfermagem e Fisioterapeutas	22	17F / 5M	26 - 55 anos	17 enfermeiros, dois técnicos de enfermagem e três fisioterapeutas. Deles, 14 atuavam no SUS em ambientes hospitalares (ambulatórios, internamentos, UTI e CP) e cinco em UCC vinculados a planos de saúde e uma em serviço particular de CP.
Total	96	75F / 21M	22 - 82 anos	Diversidade de papéis familiares e profissionais em diferentes cenários assistenciais.

Nota: F = feminino; M = masculino; CP = cuidados paliativos; SAMU = Serviço de Atendimento Móvel de Urgência; DAV = Diretivas Antecipadas de Vontade; UPA = Unidade de Pronto Atendimento; UCC = Unidades de Cuidados Continuados; SUS = Sistema Único de Saúde.

A diversidade geográfica e institucional permitiu a identificação de quatro cenários assistenciais: Cenário I (SUS com equipe de CP): atendimento multiprofissional com assistência domiciliar eventual; Cenário II (SUS sem CP estruturado): seguimento majoritário por oncologistas, com apoio da Estratégia de Saúde da Família ou do Programa Melhor em Casa; Cenário III (plano com rede própria e CP estruturado): equipe especializada, assistência domiciliar 24h e fornecimento de medicamentos; Cenário IV (plano sem estrutura de CP ou atendimento particular): cuidado centrado no oncologista, com busca autônoma por suporte complementar.

A análise dos dados dos cinco GA permitiu a construção da teoria substantiva denominada “A construção social do local de morte no câncer avançado: entre mediações e obstáculos” constituída por duas categorias: 1) Viabilizando escolhas e mediando significados: a comunicação como ponte entre desejos e realidades e, 2) Barreiras sistêmicas: obstáculos à construção de significados compartilhados. Neste artigo serão apresentadas a primeira categoria e suas subcategorias, a saber: 1) O local de morte como espaço simbólico e as 2) Dinâmicas relacionais: conflito, mediação e viabilização.

Na Figura 1 observa-se a contraposição entre hospital e domicílio como opções de “escolha” para o local de morte. Identifica-se que não há unanimidade na escolha, em realidade o hospital, para muitos, representa tanto um sentimento de segurança, controle e alívio da dor quanto de frieza e distanciamento emocional; ao passo que o domicílio concebe em determinado momemto uma ideia de respeito à autonomia, intimidade e sentimento de pertencimento, mas em oturos momentos é tido como gerador de medos e insegurança. A tomada de decisão envolve um processo de negociação e consenso modulada pela ponderação de fatores subjetivos (crenças, espiritualidade, memórias), institucionais (protocolos, cerco do silêncio) e sistêmicos (ausência de recursos, condições clínicas, sobrecarga de cuidadores, falhas de comunicação). Setas circulares evidenciam a importância do diálogo entre PCA, familiares e profissionais de saúde para mediar e viabilizar escolhas, que mesmo apropriadas em determinada situação, pode deixar de ser em outra.

**Figura 1:** Diagrama que sintetiza os processos experienciados/identificados na categoria – Maringá, PR, Brasil, 2025.

### Viabilizando Escolhas e Mediando Significados: A Comunicação Como Ponte Entre Desejos e Realidades

Nesta categoria observa-se que a comunicação desempenha papel central na mediação de significados, sendo construída a partir das interações simbólicas entre PCA, familiares e profissionais de saúde. Essa mediação possibilita a construção de significados compartilhados sobre o local de morte, com o objetivo de respeitar a autonomia e preservar a dignidade até o final da vida. Nesse contexto, identificou-se que o manejo adequado da dor e do sofrimento constitui o elemento simbólico central na modulação, ressignificação e definição de uma nova decisão, que tanto pode reafirmar a primeira, ou modificá-la.

Uma sumarização das falas e subcategorias que integram a categoria e exemplificam os achados é apresentada no Quadro 3.

**Quadro 3:** Categoria, subcategorias e falas dos participantes – PR, Brasil, 2025.

Viabilizando escolhas e mediando significados: a comunicação como ponte entre desejos e realidades	
*O local de morte como espaço simbólico*	*[...] posso mudar de ideia, mas, a princípio, o meu desejo é que seja no hospital, bem assistida, até para evitar que eles se desgastem [...] porque tenho os meninos pequenos [...]. Então, assim, no hospital tem médicos, tem equipe. Eu me sinto mais segura no hospital*. (PCA01, 41 anos) *Porque no hospital, eles têm recursos que a gente não tem. Então quando ele foi sedado, ficou tranquilo, quietinho, aí na madrugada morreu*. (F09, 62 anos, esposa) *[...]muitas vezes o paciente e a família se sentem mais seguros no hospital [...] ter recursos mais próximos como oxigênio, medicação de forma imediata. Muitas vezes acontece de ter um contexto emocional mais vulnerável [...] seja porque os familiares não gostariam de presenciar o óbito ou porque tem crianças em casa, então optam para que o paciente venha até o hospital*. (MED-15, 34 anos) *Eu quero estar em casa com as pessoas que amo, no lugar que me sinto bem, me sinto acolhida. Este é o meu pedido*. (PCA15, 39 anos) *Decidiram ficar em casa, perto da família, pelo conforto da casa e comodidade. Já não tinha muito o que fazer*. (FA06, 33 anos, filho) *O ambiente hospitalar não é aconchegante igual em casa com os familiares mais próximos. Estar ali no seu conforto, no seu cantinho*. (PS08, 35 anos) *Importante é não ter dor, estar em um ambiente mais adequado*. (PCA03, 45 anos) *Penso no final de vida estar sendo bem cuidada, porque a dor não é legal*. (PCA23, 48 anos)
*Dinâmicas Relacionais: Conflito, mediação e viabilização*	*Eu e os filhos decidimos. Eles falavam: ´pai, não pode deixar aqui, nós temos que levar´ se ela morrer lá, pelo menos estará sendo atendida pela medicina*. (FA02, 69 anos, esposo) *Começou a piorar muito a insuficiência respiratória, estava meio que agonizando. O marido chamou os três filhos e falou: a gente tinha prometido que não ia deixar morrer no hospital, que ia ficar aqui em casa com a gente, mas vendo o que ela está passando agora, não consigo deixar ela sofrer desse jeito. Se no hospital eles colocarem ao menos um cateter nasal para ela respirar até ela parar, é melhor. Aí decidiram levar, então foi numa conversa entre eles*. (FA05, 22 anos, sobrinha). *Tem situações que o paciente quer ir para casa, mas a família não quer. Para cuidar tem que ter um rodízio, tem que investir em profissionais. Então a família fala: ‘não tem como’, você vai ficar aqui no hospital*. (ENF17, 26 anos) *Se a gente a levasse para o hospital, talvez ficasse uma pessoa com ela ou ela ia ficar em uma UTI e ia morrer lá sozinha. Ela também não queria ir, por isto decidimos passar por esse sufoco, porque foi um sufoco. Minha irmã não conseguia... chegou a chamar o SAMU. Eu sou um pouco mais calma e fiquei do lado dela o tempo todo. Estava segurando sua mão na hora em que morreu. O médico do SAMU falou: “fica a critério de vocês, a gente está aqui para fazer o que vocês quiserem, só que assim... a única diferença é que aqui ela está cercada de amor, e no hospital ela vai ficar lá ..., mas se vocês quiserem a gente leva`. Daí decidimos que não ia levar”.* (FA11, 44 anos, filha)

### O Local de Morte Como Espaço Simbólico

A escolha do hospital está relacionada à segurança técnica, tendo o significado de um espaço que oferece controle eficaz da dor e do sofrimento, devido à disponibilidade imediata de recursos e suporte profissional. A escolha do hospital também é influenciada por valores culturais, crenças religiosas, experiências pessoais com os serviços de saúde e vivências relacionadas à terminalidade. Por exemplo, a partir da preocupação em mitigar o sofrimento dos familiares, principalmente das crianças ao presenciarem os momentos finais; o aumento da sobrecarga familiar nos cuidados finais; e até evitar memórias negativas no ambiente domiciliar, o que faz de o hospital passar a se constituir como o local de escolha para muitos.

Por outro lado, o domicílio é simbolizado como um lugar de dignidade e conforto e representa conexão emocional, acolhimento, intimidade, respeito à autonomia da PCA e fortalecimento de laços familiares. A busca por dignidade e autonomia está intrinsecamente relacionada ao significado atribuído à dor e ao sofrimento, sendo o manejo eficaz desses fatores o principal determinante na tomada de decisão. Quando a dor e o sofrimento não podem ser adequadamente controlados, o domicílio deixa de ser uma opção viável, evidenciando o distanciamento do conforto desejado.

Outro aspecto relevante é que o manejo adequado da dor simboliza dignidade e respeito nos momentos finais da vida. Esse manejo transcende o controle físico, alcançando dimensões emocionais e simbólicas, o que influencia diretamente a percepção de dignidade e a viabilidade do local escolhido para ocorrer a morte. Quando o controle da dor e do sofrimento é inadequado, a percepção de dignidade é comprometida, e o hospital é ressignificado como espaço de alívio e segurança.

### Dinâmicas Relacionais: Conflito, Mediação e Viabilização

A busca pelo alívio ou privação da dor e do sofrimento desencadeia um processo de negociação sobre o local de morte, envolve as pessoas com câncer, seus familiares e os profissionais de saúde. Essa dinâmica, marcada por conflitos, negociações, mediações e viabilização é permeada pela comunicação, que atua como elemento central para alinhar expectativas e facilitar a tomada de decisão.

As dinâmicas familiares ilustram o *Self* coletivo, no qual os significados são negociados entre os membros. Nota-se ainda que, os conflitos decisórios podem ocorrer por divergências entre os desejos da PCA e dos seus familiares. De forma geral, os conflitos emergem de diferentes interpretações sobre o que é “melhor” para a PCA. Esses conflitos são momentos de negociação simbólica, sendo os significados ajustados ou reafirmados por meio da mediação. Os agentes mediadores desse processo de ressignificação contínua podem ser os familiares ou os profissionais de saúde.

O *Self* dos familiares também é moldado na interação, ao assumirem papéis de cuidado, negociação e tomada de decisão. O conflito entre familiares pode ser interpretado como uma luta simbólica pela preservação do *Self* coletivo dos familiares. Nestes casos, os profissionais de saúde têm papel crucial na facilitação dessa mediação, sendo essenciais na ressignificação de medos e expectativas ao viabilizarem novas experiências que possam ressignificar o local de morte, e ajudando assim os familiares a alinharem os significados e expectativas. Essa mediação não só resolve conflitos, mas também cria significados compartilhados sobre o local de morte.

## DISCUSSÃO

Os resultados evidenciam a importância da mediação profissional, da escuta ativa e da comunicação eficaz para favorecer a negociação de significados e decisões compartilhadas entre pacientes, familiares e equipes de saúde - aspectos identificados como fundamentais para a promoção de cuidados centrados na pessoa e na qualidade de vida na terminalidade. Estudos prévios destacam que a comunicação assertiva e o envolvimento dos profissionais de enfermagem no processo decisório são estratégias cruciais para garantir a autonomia do paciente e fortalecer a relação terapêutica no contexto de cuidados paliativos, contribuindo para práticas mais humanizadas e alinhadas aos valores individuais dos pacientes^(19,20)^.

A categoria “Viabilizando escolhas e mediando significados: a comunicação como ponte entre desejos e realidades” mostra que a decisão sobre o local de morte constitui-se num processo simbólico, interacional e relacional. Este estudo amplia a compreensão do fenômeno ao evidenciar que símbolos como hospital, domicílio, dor e dignidade integram os significados atribuídos ao local de morte, os quais são constantemente negociados e viabilizados ou inviabilizados diante das condições estruturais e relacionais disponíveis. Esse conhecimento oferece subsídios teóricos para práticas e políticas em cuidados paliativos, o que é reforçado por investigações que demonstram que elementos simbólicos orientam tanto familiares quanto equipes multiprofissionais na coconstrução desses significados^(21,22)^.

Os significados atribuídos ao local de morte emergem das relações sociais e são moldados pela maneira como os sujeitos interpretam suas experiências^(12)^. Assim, o hospital é simbolizado como espaço de proteção e cuidado técnico ou, inversamente, como lugar de distanciamento e ruptura com a dignidade, conforme revelado neste e em outros estudos^(3,6)^. Sendo assim, o local de morte adquire sentidos diversos conforme o repertório simbólico que o paciente e seus interlocutores constroem ao longo da trajetória da doença.

Em contextos não oncológicos, resultados semelhantes também foram identificados. Estudos internacionais^(6,8,23)^ apontam que, em doenças como demência ou insuficiência renal crônica, o hospital tende a ser o local predominante de morte, mas em descompasso com as preferências previamente expressas, sobretudo pelo insuficiente suporte domiciliar e pela sobrecarga familiar. Isso reforça que a construção social do local de morte - ora como espaço de dignidade e vínculo, ora como cenário de ruptura e institucionalização - não é exclusiva do paciente com câncer, mas atravessa diferentes processos de adoecimento.

No Brasil, estudos^(7,24)^ indicam a predominância do hospital como local de morte para pessoas com câncer, sobretudo diante da piora clínica ou da falta de suporte domiciliar^(7)^. No presente estudo, significou também proteção familiar, especialmente para evitar que crianças testemunhassem a terminalidade, prevenindo impactos emocionais e aliviando a sobrecarga dos entes queridos. Por outro lado, o domicílio simbolizava respeito à dignidade e autonomia, além de manutenção de conexão emocional, proporcionada pela proximidade com entes queridos^(6)^ e preservação do *Self*. Contudo, quando os familiares não se sentem preparados para enfrentar a evolução natural dos cuidados no fim de vida, passando a vivenciar grande sofrimento, essa preferência pode ser ressignificada, tornando o hospital uma escolha inevitável^(5)^. Logo, a morte em casa representa não apenas uma escolha de local, mas uma afirmação do *self* em seu ambiente afetivo, mostrando que a geografia do cuidado é, antes, uma narrativa de identidade.

Entretanto é preciso considerar que diferenças regionais na disponibilidade de serviços especializados e a dificuldade de acesso podem influenciar a escolha do local de morte. Para além, de acordo com alguns relatos, no contexto do câncer, são marcantes as diferenças no acesso e disponibilidade de serviços, inclusive dentro da mesma região/estado, a depender do local de moradia – se capital ou interior / se área urbana ou rural. Este fato certamente tem implicações importantes para a equidade em cuidados paliativos. Estudo sobre a tendência temporal da mortalidade por neoplasia no Brasil entre 2002 e 2022, constatou por exemplo, que São Paulo e Rio Grande do Sul apresentaram altas taxas de óbitos no ambiente hospitalar, possivelmente associadas à maior oferta de serviços de referência oncológica. Por outro lado, estados do Norte e Nordeste, como Maranhão e Piauí, registraram tendências crescentes de óbitos domiciliares, o que pode estar relacionado à baixa cobertura de serviços especializados^(24)^.

À despeito destas questões, evidências indicam que políticas públicas robustas, que garantem acesso, escuta e suporte contínuo, aumentam a possibilidade de alinhar o local de morte às preferências do paciente, promovendo dignidade, conforto e redução de intervenções desnecessárias no final da vida^(25,26)^. Desse modo, a PNCP instituída no Brasil em 2024^(10)^, representa um avanço importante para a superação das barreiras estruturais, ampliando o acesso ao cuidado domiciliar, fortalecendo equipes multiprofissionais e alinhando-se a modelos internacionais bem- sucedidos^(26)^. Para tanto será necessário que a Atenção Primária Saúde se reestruture com equipes de cuidados paliativos para que pacientes e familiares possam contar com o suporte no manejo da dor e sofrimento evitando com isso a necessidade da busca pelo hospital no momento de agudização do sofrimento^(27)^.

A PNPC visa o desenvolvimento de ações que estimulem à educação em CP com incentivo à formação e educação continuada dos profissionais das equipes que atuam na Rede de Atenção à Saúde^(28)^, da qual a equipe de enfermagem tem um papel preponderante na integração entre os demais membros da equipe e a unidade de cuidados paciente e familiares.

Adicionalmente, a construção do *self* no processo de terminalidade, compreendida como a forma como a pessoa se vê e é reconhecida pelos outros^(29)^, é mobilizada constantemente. O desejo de permanecer em casa expressa a intenção de manter a continuidade do *self* diante da finitude; ao passo que a hospitalização, ainda que restrinja o controle, pode simbolizar proteção e esperança de amparo diante do sofrimento. Isso reitera que as preferências oscilam conforme a negociação entre valores pessoais e necessidades clínicas.

A mediação profissional emerge como elemento chave na reconstrução de significados. Os profissionais que escutam e acolhem, ampliam possibilidades ao atuarem como mediadores simbólicos do processo de morrer^(29)^. Ao assumir o papel do outro^(30)^, esses profissionais favorecem a expressão do *self* e contribuem para uma decisão compartilhada, ancorada em valores e desejos reais. Destarte, os conflitos familiares emergiram como um aspecto no processo decisório sobre o local de morte e destacam a importância do papel do enfermeiro não apenas como cuidador clínico, mas também como mediador comunicacional entre os membros da família. A literatura em cuidados paliativos ressalta que profissionais de enfermagem podem operacionalizar técnicas de mediação familiar por meio de estratégias como encontros familiares estruturados, uso de comunicação empática e facilitadora, escuta ativa e orientação para a resolução de divergências de valores e expectativas, promovendo entendimento compartilhado e decisões conjuntas sobre o cuidado^(31)^. Essas abordagens podem contribuir para reduzir tensões, fortalecer o suporte emocional e alinhar as escolhas sobre o local de morte com as preferências dos pacientes e de seus familiares, integrando habilidades clínicas e comunicacionais no cuidado holístico.

Ademais, é necessário reconhecer as limitações marcadas por conflitos familiares insolúveis, estruturas precárias e resistência institucional à escuta ativa, que constituem limites éticos e contextuais que tensionam essa mediação e impactam na viabilização do local de morte desejado, momento em que cabe a realização de uma conferência familiar, como instrumento terapêutico em prol da resolução de conflitos e dilemas éticos^(32)^.

Nesse contexto, a conferência familiar é uma intervenção terapêutica, mediada por profissionais da equipe de cuidados paliativos, se constituindo num espaço estruturado de comunicação que favorece a escuta ativa, a expressão de valores e a negociação de expectativas entre pacientes, familiares e equipe de saúde, reduzindo conflitos e promovendo decisões compartilhadas. Ao atuar como mediador comunicacional, o profissional, que em muitos momentos é o enfermeiro, contribui para alinhar as escolhas sobre o cuidado - incluindo o local de morte - às preferências do paciente, fortalecendo o entendimento mútuo e o cuidado centrado na pessoa^(32)^.

A interação entre pacientes, familiares e profissionais de saúde pode desencadear conflitos simbólicos que exigem comunicação eficaz e busca de consenso. Em contextos como a China, o “cerco do silêncio” - ocultação da condição para proteção emocional - limita a autonomia e dificulta decisões compartilhadas^(33)^, e cenários similares se reproduzem no Brasil, agravados pela fragmentação dos serviços e pela ausência de políticas integradas^(7)^.

Além disso, as instituições de saúde enfrentam desafios na comunicação sobre o morrer e a morte, uma vez que modelos biomédicos ainda predominam e a linguagem técnica frequentemente obscurece a escuta e a expressão dos desejos da PCA^(9,34)^. Nesse ambiente, o “cerco do silêncio”, embora culturalmente motivada, prejudica a participação ativa do paciente no processo decisório^(30,33)^, rompendo a construção compartilhada de significados e impedindo o *self* de se expressar diante da finitude.

A interação institucional reduz a morte a um evento técnico, negando sua dimensão simbólica, afetiva e relacional. Além de que, não apenas obscurecem ou distorcem os significados atribuídos pela PCA e seus familiares, mas também contribuem para a ausência de um planejamento prévio adequado para os momentos finais da vida. Portanto, comunicação clara e empática entre profissionais, pacientes e familiares é fundamental para mitigar barreiras e facilitar decisões^(5,8)^. Porém, a mediação profissional, embora desejável e potente, depende de estrutura, tempo, preparo emocional e legitimidade institucional.

Diante desse desafio comunicacional, o enfermeiro - enquanto mediador simbólico e profissional de referência no cuidado cotidiano - deve aprimorar suas habilidades de escuta terapêutica e comunicação segura. Para tanto, precisa saber identificar o momento oportuno para abordar as dúvidas sobre o processo de morrer e morte com o paciente e seus familiares^(35)^, de modo a restabelecer a interação horizontal e a coconstrução de significados no processo decisório.

A Resolução nº 564/2017 do Código de Ética dos Profissionais de Enfermagem destaca a responsabilidade do enfermeiro em promover condições para a autonomia do paciente na tomada de decisões sobre cuidado e conforto^(36)^. Em âmbito internacional, o Código de Ética do Conselho Internacional de Enfermeiros^(37)^ também reforça o respeito à dignidade humana, aos direitos e às escolhas dos pacientes em todas as etapas da vida, incluindo o fim de vida.

Esses referenciais éticos, nacionais e internacionais, evidenciam que a prática da enfermagem deve ser pautada por uma atuação humanizada, sensível à singularidade de cada pessoa e promotora da autonomia, independentemente do local de atenção^(38)^. Nesse contexto, a equipe de enfermagem se configura como mediadora simbólica, uma vez que a escolha do local de morte demanda não apenas infraestrutura, mas também relações horizontais, escuta ativa e ambientes simbolicamente acolhedores, permitindo a realização de ações personalizadas^(20)^ que atendam às necessidades específicas e valorizem o “hoje” com suas limitações e possibilidades, promovendo qualidade aos dias que restam.

O reconhecimento de que a escolha do local de morte é uma construção simbólica impõe que, além de infraestrutura, seja assegurado às pessoas o poder de atribuir sentido a essa decisão por meio de interações horizontais e ambientes acolhedores^(5)^. Os modelos de atenção que desconsideram linguagem, valores e dinâmicas sociais tendem a reproduzir desigualdades e práticas desumanizantes, enquanto uma abordagem centrada nos significados pode fortalecer a eficácia clínica, a justiça social e o cuidado ético no fim da vida. Portanto, reconhecer o caráter relacional e simbólico dessas decisões orienta práticas e políticas que valorizam o protagonismo dos pacientes, promovem a equidade e possibilitam uma assistência verdadeiramente humanizada nos cuidados paliativos.

Dentre as possíveis limitações do estudo cita-se as entrevistas retrospectivas com familiares que podem envolver reconstruções subjetivas dos eventos finais. Além disso, a predominância de entrevistas remotas - que por um lado ampliaram o alcance geográfico - pode ter limitado a percepção de nuances não verbais. Por fim, a concentração de participantes oriundos de áreas urbanas e do sexo feminino, não permitiu que realidades rurais e diferentes perspectivas de gênero fossem capturadas. Assim, considerando que os significados atribuídos ao local de morte são negociados e viabilizados ou inviabilizados diante das diferentes condições estruturais e relacionais, acredita-se que a valorização das nuances do contexto urbano e rural e a participação de um número maior de pessoas do sexo masculino, sobretudo entre PCA e familiares, pode enriquecer a compreensão do fenômeno. Aponta-se, portanto, para a necessidade de pesquisas futuras em contextos assistenciais diversos.

## CONCLUSÃO

A teoria substantiva construída descreve a decisão sobre o local de morte como um processo simbólico, interacional e dinâmico. A análise das narrativas revelou camadas de significados negociados em torno do hospital e do domicílio, influenciadas por valores culturais, experiências prévias, condições institucionais e recursos disponíveis. Essa abordagem possibilitou compreender como esses significados são construídos, ressignificados e compartilhados, permitindo identificar fatores que moldam essa decisão no contexto oncológico.

A escolha do local de morte se configura como expressão do *self* mediada pela linguagem, escuta ativa e capacidade de assumir o papel do outro. A interdependência entre paciente, familiares e equipe multiprofissional - em especial o papel do enfermeiro como mediador simbólico - é fundamental para garantir tempo, acolhimento e dignidade. As implicações para a prática e para as políticas públicas incluem o investimento em cuidados domiciliares, apoiados na PNCP. Futuros estudos longitudinais devem comparar as realidades antes e depois da implementação da PNCP para avaliar seu impacto na viabilização de escolhas, mediação de significados e superação de barreiras sistêmicas.

## Data Availability

Todo o conjunto de dados que dá suporte aos resultados deste estudo está disponível mediante solicitação ao autor correspondente.

# Translation: Proceso relacional y simbólico: la decisión sobre el lugar de la muerte en el contexto del cáncer avanzado*

**DOI:** 10.1590/1980-220X-REEUSP-2025-0405es

**Authors:** Patrícia Chatalov Ferreira, Marcelle Miranda da Silva, Mayckel da Silva Barreto, Esperanza Begoña Garcia-Navarro, Cristina Garcia-Vivar, Eleandro do Prado, Rudval Souza da Silva, Sonia Silva Marcon

**Affiliations:** 1 Universidade Estadual de Maringá, Programa de Pós-Graduação em Enfermagem, Maringá, PR, Brasil.; 2 Universidade Federal do Rio de Janeiro, Programa de Pós-Graduação em Enfermagem, Rio de Janeiro, RJ, Brasil.; 3 Universidade de Huelva, Departamento de Enfermagem, Huelva, España.; 4 Universidad Pública de Navarra, Departamento de Enfermagem, Pamplona, Navarra, España.; 5 Universidade Positivo, Colegiado de Enfermagem, Curitiba, PR, Brasil.; 6 Universidade do Estado da Bahia, Programa de Pós-Graduação em Ciências do Cuidar em Saúde, Senhor do Bonfim, BA, Brasil.

**Keywords:** Cuidados Paliativos, Neoplasias, Toma de Decisiones, Familia, Enfermería, Teoría Fundamentada.

## Abstract

**Objetivo:**

Construir una teoría sustantiva sobre los significados atribuidos y los factores que influyen en la decisión sobre el lugar de la muerte en el contexto del cáncer avanzado.

**Metodología:**

Se utilizaron como marcos de referencia el interaccionismo simbólico y la teoría fundamentada en los datos. Se realizaron entrevistas grabadas en audio, presenciales o a distancia, entre 2023 y 2024, con 96 participantes de nueve estados ubicados en cuatro regiones brasileñas, que integraron cinco grupos de muestra.

**Resultados:**

El hospital representa un espacio de protección, alivio del dolor y del sufrimiento/sobrecarga familiar, y el domicilio, un entorno que promueve la dignidad y la autonomía del paciente y la conexión afectiva con los familiares, acompañado de inseguridad respecto al manejo del dolor y el sufrimiento. La mediación profesional y la escucha activa favorecen la negociación de significados, mientras que las barreras estructurales y comunicacionales limitan la viabilidad de las elecciones.

**Consideraciones Finales:**

La decisión sobre el lugar de muerte constituye un proceso simbólico, relacional y dinámico, moldeado por conflictos familiares, limitaciones en la estructura física e interacciones entre pacientes, familiares y el equipo multiprofesional. Es importante la implementación de políticas públicas que permitan elecciones coherentes con los valores de la persona con enfermedad terminal.

## INTRODUCCIÓN

La decisión sobre el lugar de muerte en el contexto del paciente con cáncer avanzado se ha convertido en una cuestión central, especialmente ante el aumento progresivo de las neoplasias y las afecciones crónicas complejas, lo que intensifica los desafíos para los sistemas de salud en países en desarrollo, como Brasil^(1)^. La pandemia de COVID-19 agravó esta realidad, retrasando los diagnósticos y comprometiendo el acceso a los servicios, lo que contribuyó a la identificación tardía de casos en estadio avanzado^(2)^.

Enfrentarse a una afección irreversible, aunque no implique la muerte inmediata, exige decisiones complejas relacionadas con los cuidados al final de la vida. Entre ellas, destacan la definición del entorno en el que se desea que ocurra la muerte, la elección del lugar para los cuidados paliativos y la organización de la atención^(3)^. La noción pragmática de “muerte suficientemente buena” (*good enough death*) se centra en la oferta de una atención adecuada y en el control de los síntomas físicos, emocionales y espirituales. Este cambio explicaría la divergencia entre las expectativas difícilmente alcanzables de una propuesta de muerte idealizada y la realidad cotidiana del trabajo en los servicios, marcada por la complejidad y la heterogeneidad^(4)^. De este modo, las decisiones sobre el lugar donde ocurrirá la muerte están atravesadas por factores clínicos, emocionales, sociales y simbólicos, lo que explica la diversidad de preferencias y motivaciones que orientan las elecciones^(5)^. Por lo tanto, comprender este proceso de toma de decisiones requiere considerar no solo aspectos objetivos, sino también los significados construidos en las interacciones sociales.

Gran parte de los estudios que abordan la elección del lugar de muerte se han llevado a cabo en países desarrollados, presentando enfoques limitados que apenas tienen en cuenta la naturaleza interactiva del proceso de toma de decisiones^(3,5,6)^. Esta brecha de conocimiento cobra aún más relevancia cuando se observa en el contexto brasileño, marcado por las desigualdades en el acceso a los servicios de salud y la diversidad cultural, factores que hacen que este debate sea aún más complejo^(3,7)^.

En la literatura nacional e internacional, los estudios cuantitativos relacionan la elección del lugar de muerte con características sociodemográficas, como la edad, el estado civil y el apoyo familiar^(3,5,6,7,8)^. Sin embargo, siguen siendo escasas las investigaciones cualitativas que exploren en profundidad los significados atribuidos y las interacciones sociales que sustentan este proceso^(9)^. Esta carencia de estudios no solo dificulta una comprensión más amplia y contextualizada del fenómeno, sino que también limita la producción de marcos teóricos y prácticos capaces de orientar políticas y prácticas profesionales más sensibles a la realidad vivida por las personas. En Brasil, esta escasez es aún más evidente en las revistas del área de enfermería, lo que refuerza la necesidad de estudios que amplíen la comprensión de este fenómeno desde una perspectiva interaccional y práctica^(7)^.

En el contexto de Brasil, las investigaciones sobre el proceso de toma de decisiones respecto al lugar de muerte siguen siendo incipientes y fragmentadas, sobre todo si se considera la complejidad relacional que envuelve el trípode formado por pacientes, familiares y equipos de salud. La originalidad de este estudio reside en el enfoque comprehensivo e interactivo adoptado, que privilegia la construcción de significados compartidos y las dinámicas comunicacionales implicadas en este proceso. Al proponer una teoría sustantiva basada en evidencia empírica, el trabajo amplía la comprensión del fenómeno más allá de las variables individuales, ofreciendo una lectura innovadora que integra las dimensiones subjetivas, sociales e institucionales de los cuidados al final de la vida^(7)^.

Además, cabe destacar que la presente investigación también se sustenta en consonancia con las directrices de la Política Nacional de Cuidados Paliativos (PNCP)^(10)^, la cual enfatiza la centralidad de la persona y la familia en las decisiones sobre los cuidados, así como la necesidad de ampliar el acceso equitativo a servicios de calidad en todos los niveles de atención, especialmente en un país como Brasil, en el que la clasificación sobre la calidad de los cuidados paliativos lo sitúa en el puesto 79 de una lista de 81 países^(11)^. Comprender los factores y significados que orientan la decisión sobre el lugar de muerte contribuye directamente a la implementación de esta política, al propiciar prácticas asistenciales más humanizadas, comunicativas y culturalmente sensibles. Además, se puede suponer que los hallazgos apoyarán a los gestores y profesionales en la formulación de estrategias orientadas a la organización de las redes de atención, fortaleciendo los cuidados paliativos como componente esencial del sistema de salud y reafirmando el papel de la enfermería en la coordinación y la integralidad de los cuidados al final de la vida.

Ante esta brecha de conocimiento, el objetivo del estudio fue construir una teoría sustantiva sobre los significados atribuidos y los factores que influyen en la decisión sobre el lugar de muerte en el contexto del cáncer avanzado. Esta comprensión tiene implicaciones directas para la práctica de la enfermería oncológica y para la organización de los servicios de cuidados paliativos, al favorecer estrategias de atención más sensibles a las necesidades de las personas con cáncer avanzado y sus familiares.

## MÉTODO

### Diseño del Estudio y Marco Teórico y Metodológico

Se trata de una investigación cualitativa, de carácter explicativo, que utilizó como marco teórico el Interaccionismo Simbólico (IS), según el cual los significados atribuidos a las situaciones o cosas surgen de las interacciones sociales y de las experiencias vividas^(12)^. La Teoría Fundamentada en los Datos (TFD), de orientación constructivista, fue la opción metodológica. Esta orientación presupone que las experiencias tanto del investigador como de los participantes son relevantes e influyen en la interpretación de los datos y, por consiguiente, en la construcción del fenómeno^(13)^. En la elaboración del informe de investigación se siguieron las directrices *Consolidated Criteria for Reporting Qualitative Research* (COREQ).

### Lugar, Población y Criterios de Selección

Participaron en el estudio 96 personas, distribuidas en cinco grupos de muestra (GM) constituidos por familiares de personas fallecidas por cáncer hace como máximo un año, personas con cáncer avanzado, médicos, psicólogos y asistentes sociales y, por último, enfermeros y fisioterapeutas.

Los criterios de inclusión para pacientes y familiares fueron tener 18 años o más y capacidad de comunicación verbal. No se incluyeron individuos con inestabilidad emocional o sin condiciones para acceder a recursos tecnológicos, en el caso de las entrevistas a distancia. En cuanto a los profesionales, se consideró que tuvieran al menos seis meses de experiencia en la atención a personas con cáncer.

### Muestreo Teórico

La recolección de datos comenzó con cuatro familiares de personas fallecidas a causa del cáncer, indicados por profesionales de una institución filantrópica que opera en el municipio sede del estudio. Al final de las entrevistas se solicitó la indicación de nuevos participantes potenciales, tal como propone la técnica de muestreo no probabilístico denominada *Snowball Sampling* (bola de nieve)^(14)^. Sin embargo, hubo dificultades para lograr la participación de las personas indicadas y, considerando que las redes sociales se han utilizado de manera satisfactoria como escenario de estudios cualitativos, incluso al abordar temas delicados como la violencia doméstica^(15)^, se optó por explorar otros contextos culturales y socioeconómicos, reclutando a familiares y personas con cáncer avanzado que compartían sus experiencias utilizando las redes sociales como recurso.

Los estudios incluidos en una revisión^(16)^ indican que el reclutamiento de participantes a través de las redes sociales es una estrategia eficaz y de bajo costo en la investigación en salud, siempre que vaya acompañada de criterios rigurosos de verificación de la credibilidad, como la selección previa, el cruce de información y los mecanismos para identificar perfiles inadecuados. Así, con el fin de mitigar los riesgos éticos, la selección de estos participantes se llevó a cabo de manera minuciosa, considerando, además de los aspectos mencionados, la mediación de los administradores de los grupos y la confirmación de la elegibilidad durante el contacto inicial, de acuerdo con las recomendaciones metodológicas para la Recolección remota de datos cualitativos^(17)^. Además, los riesgos éticos inherentes a este tipo de reclutamiento se mitigaron mediante la obtención del consentimiento informado, la garantía de confidencialidad, la atención a la privacidad de los participantes y el monitoreo continuo de posibles malestares emocionales, en consonancia con la literatura especializada^(18)^.

El primer grupo de muestra (GM) estuvo compuesto por 15 familiares de personas fallecidas a causa del cáncer (cinco en entrevistas presenciales y 10 en entrevistas *en* línea). El análisis de los datos de este grupo mostró que la decisión sobre el lugar de muerte está influenciada por las dinámicas familiares y la comunicación con los profesionales de la salud, marcada por el “Cerco del Silencio” y por un “Modelo Paternalista”. Así surgió la hipótesis de que las estrategias de “protección” adoptadas por los familiares pueden interferir en la autonomía de la persona con cáncer avanzado (PCA) en relación con la decisión sobre el lugar de la muerte. De este modo, se definió la necesidad de incluir a estas personas y escucharlas en relación con la autonomía en la elección del lugar de la muerte.

Así, el segundo grupo de muestra (GM) estuvo compuesto por 24 PCA, identificadas en perfiles de Instagram que mencionaban en sus biografías o publicaciones públicas términos como “cáncer avanzado”, “metástasis”, “metastático”, “paliativo” y “paliativa”. Las invitaciones para participar se enviaron mediante mensajes directos, y cuando se aceptaban, se programaba el día y la hora para realizar la entrevista por videollamada en Google Meet. La pregunta guía en este grupo fue “Cuéntame tu historia con el cáncer”. El tema de la elección del lugar de muerte solo se abordó en el momento oportuno, después de que la investigadora percibiera una disposición para ello, con el fin de no desencadenar desencadenantes ni sufrimiento emocional.

Durante la recolección y el análisis de las entrevistas se constató que las personas con cáncer (PCA) mostraban preferencia sobre el lugar de muerte; sin embargo, se percibió que había personas que no tenían un espacio para hablar de esto con sus familiares o con profesionales de la salud, especialmente con su médico. Este contexto dio lugar a la siguiente pregunta: ¿Cuál es la opinión de los médicos sobre la elección del lugar de muerte de los pacientes con cáncer? La hipótesis era que los médicos creen que los pacientes con cáncer no están en condiciones de tomar esta decisión.

De este modo, el tercer grupo de muestra (GM) estuvo integrado por 20 médicos. La pregunta guía fue: “Háblenme sobre las decisiones relativas al lugar de muerte de los pacientes con cáncer a los que atiende o ha atendido”. El análisis de los datos de este grupo mostró que los oncólogos y los especialistas en cuidados paliativos abordan de diversas maneras las cuestiones sobre la muerte, los cuidados paliativos y el lugar de muerte con los familiares y con el propio paciente en fase terminal. Surgió así la siguiente pregunta: ¿Cuál es el papel de otros profesionales de la salud en el proceso de toma de decisiones? La hipótesis era que el apoyo psicológico a los pacientes y familiares influía en la aceptación de la terminalidad y en la decisión sobre el lugar de muerte.

Por consiguiente, el cuarto grupo de muestra (GM) estuvo integrado por 15 profesionales, de los cuales 11 eran psicólogos y cuatro, asistentes sociales. Se observó que los primeros ayudan a los médicos en la comunicación difícil y trabajan directamente con las dimensiones emocionales y psicológicas relacionadas con la terminalidad, mientras que los asistentes sociales se mostraron fundamentales en el apoyo a los familiares, ocupándose de cuestiones logísticas, sociales y financieras.

A partir de los datos obtenidos en este grupo, se observó que los factores sociales y económicos influyen fuertemente en la decisión sobre el lugar de muerte y que el apoyo logístico hace posible la atención al final de la vida en el hogar.

Además, algunos relatos de los pacientes con Alzheimer y de sus familiares destacaron que los enfermeros se involucran directamente en la prestación de cuidados y en el apoyo emocional, tanto en el hospital como en el hogar, además de actuar con frecuencia como mediadores entre médicos y familiares/pacientes, y que los fisioterapeutas ayudaban a proporcionar comodidad física y a enseñar mejores estrategias para la movilidad del paciente. La pregunta que surgió fue: ¿Cómo perciben estos profesionales la influencia de su actuación en la decisión sobre el lugar de muerte del paciente terminal? Por lo tanto, el quinto y último grupo de muestra (GM) estuvo compuesto por 22 profesionales, de los cuales 17 eran enfermeros, dos técnicos de enfermería y tres fisioterapeutas. En el Cuadro 1 se presentan, a modo ilustrativo, ejemplos de preguntas utilizadas con los integrantes de los cinco grupos de muestra, las cuales se ajustaron de acuerdo con el análisis comparativo constante.

**Cuadro 1:** Ejemplos de preguntas adicionales utilizadas en los cinco grupos de muestra – PR, Brasil, 2025.

Grupo	Composición	Preguntas adicionales en cada grupo
1	Familiares de personas fallecidas a causa del cáncer	¿Dónde falleció su familiar (en casa, en el hospital...)? ¿Cómo ocurrió? ¿Llegaron a discutir dónde sería mejor que ocurriera? ¿Quién participó en esa discusión? Hoy, después de que haya pasado un tiempo, ¿cuál es su opinión sobre esa decisión? ¿Por qué? Hable sobre ello.
2	Personas con cáncer avanzado	¿Alguna vez ha pensado o hablado sobre dónde (lugar) le gustaría que le cuidaran al final de su vida? ¿Con quién ha hablado? ¿Cuál es su opinión sobre la elección del lugar de fallecimiento de un familiar? ¿Quién debe decidir sobre esto? ¿Por qué? Hable más sobre esto.
3	Médicos	Según su experiencia, ¿cómo percibe que reaccionan los pacientes en cuidados paliativos y sus familiares ante la elección del lugar donde fallecer? ¿Se toma una decisión al respecto? En caso afirmativo, ¿quién participa en ella? ¿Qué factores influyen en estas decisiones en la práctica asistencial? Cuénteme más sobre las decisiones relativas al lugar de fallecimiento de los PCA a los que atiende o ha atendido.
4	Psicólogos y trabajadores sociales	¿Me puede hablar sobre el lugar de fallecimiento de los PCA a los que atiende o ha atendido? ¿En algún momento se le ha pedido que aclare dudas o que ayude en la decisión sobre el lugar de fallecimiento? ¿Cómo percibe su papel junto a estas personas y sus familiares en relación con la decisión sobre el lugar de fallecimiento? Cuénteme más sobre esto.
5	Enfermeros, auxiliares de enfermería y fisioterapeutas	Las mismas preguntas planteadas a los miembros del grupo 4, pero centradas en el contexto de la atención directa.

### Recolección de Datos

Los datos se recolectaron mediante entrevistas abiertas, grabadas en audio tras obtener la autorización de los participantes, realizadas entre febrero de 2023 y noviembre de 2024, ya sea en persona (30 personas) o por videollamada (66 personas). Las entrevistas tuvieron una duración de entre 20 y 90 minutos y todas fueron conducidas por la investigadora principal, una enfermera con experiencia en el acompañamiento de personas con cáncer y en la realización de entrevistas cualitativas, quien no tenía ningún tipo de relación con los participantes del estudio.

Durante todo el proceso de recolección y análisis de los datos, la investigadora empleó la reflexividad y la sensibilidad teórica, mediante la elaboración de notas analíticas y reflexivas después de las entrevistas y durante las etapas de codificación. Estos registros permitieron explicitar, monitorear y mantener en suspenso las preconcepciones y experiencias profesionales relacionadas con los cuidados paliativos y el final de la vida. De manera más concreta, se ejemplifica que los investigadores tenían una percepción empírica de que la ocurrencia de la muerte en casa favorecería una muerte suficientemente buena. Sin embargo, los datos también señalaban que el entorno hospitalario podría constituir un lugar de elección para la muerte por parte de algunos pacientes en cuidados paliativos y sus familias. En este sentido, los investigadores se dieron cuenta de que sus prejuicios no podían prevalecer frente a lo que revelaban los datos, evitando su incorporación acrítica en el análisis y permitiendo que las categorías surgieran a partir de los hallazgos, de acuerdo con el método de la comparación constante^(13)^.

### Tratamiento, Análisis de Datos y Validación Teórica

Las entrevistas fueron transcritas íntegramente sin el uso de *software*, por la misma investigadora y, preferentemente, el mismo día de su realización, lo que permitió enriquecer los registros con recuerdos de comportamientos no verbales. El análisis de los datos siguió las etapas de codificación inicial y focalizada, con el apoyo del *software* Max QDA^®^, que se utilizó como herramienta auxiliar en la organización de los datos, sin interferir en el proceso analítico. En la codificación inicial, la investigadora principal segmentó los datos en unidades más pequeñas, analizándolas palabra por palabra, línea por línea e incidente por incidente, lo que permitió una exploración detallada de los significados y las experiencias de los participantes. Este procedimiento permitió identificar patrones y variaciones, lo que favoreció una comprensión profunda del fenómeno investigado.

Todo el proceso analítico fue realizado inicialmente por la investigadora principal, quien se reunía mensualmente con la investigadora sénior del grupo para presentar y discutir los hallazgos. En ese momento, se exploraban las divergencias hasta llegar a un consenso.

En la codificación focalizada, los conceptos se refinaron progresivamente y adquirieron mayor relevancia analítica, lo que dio lugar a la aparición de subcategorías y categorías, y a la identificación de la categoría central, que posee mayor poder explicativo y organiza la estructura teórica del análisis: el proceso de toma de decisión sobre el lugar de muerte en el contexto oncológico. Durante este proceso fue posible identificar la saturación teórica, caracterizada por la ausencia de hallazgos que permitieran la abstracción y el avance en la teorización, mediante la identificación de nuevas *perspectivas* teóricas o de nuevas propiedades para las categorías teóricas centrales^(13)^.

Los memorandos y diagramas analíticos respaldaron la construcción de la teoría sustantiva, la cual pasó por un proceso de validación constituido por dos etapas: retorno a los datos brutos y evaluación por parte de diez individuos, siendo dos PCA, dos familiares de personas fallecidas por cáncer y seis profesionales especializados en cuidados paliativos (una psicóloga, un fisioterapeuta, tres enfermeros y un médico especialista en cuidados paliativos). En esta ocasión se identificó que no era necesario realizar modificaciones sustanciales en la categoría central, dado que los nuevos entrevistados se reconocían en el modelo teórico presentado.

### Aspectos Éticos

El estudio cumplió con las normas nacionales e internacionales de investigación con seres humanos, respetando incluso las Orientaciones para Procedimientos en Investigaciones en Cualquier Etapa en Ambiente Virtual, y fue aprobado por el Comité de Ética en Investigación con Seres Humanos de la institución signataria (CAAE 71635923.8.0000.0104; Dictamen n.º 6.224.766).

Todos los participantes fueron previamente informados sobre el objetivo de la investigación y manifestaron su consentimiento para participar, mediante la firma del Término de Consentimiento Libre e Informado, de manera presencial o virtual. Para garantizar el anonimato, en la presentación de los resultados, los extractos de los testimonios se identifican con las letras FA, PCA, MED, PS, AS, ENF, TE y FISIO para designar, respectivamente: familiar, persona con cáncer avanzado, médico, psicólogo, asistente social, enfermero, técnico de enfermería y fisioterapeuta, seguidas de un número indicativo del orden de realización de la entrevista dentro del grupo respectivo.

## RESULTADOS

Los 96 participantes del estudio procedían de nueve estados brasileños situados en cuatro regiones (Sur, Sudeste, Centro-Oeste y Nordeste). Predominaban las mujeres (75 mujeres/21 hombres), con edades comprendidas entre los 22 y los 82 años. Los participantes eran pacientes con cáncer, familiares de personas fallecidas por cáncer y profesionales de diferentes categorías del ámbito sanitario, todos residentes en zonas urbanas. Esta diversidad de perfiles permitió obtener múltiples perspectivas sobre el proceso de decisión respecto al lugar donde se produciría la muerte (Cuadro 2).

**Cuadro 2:** Caracterización de los participantes según los grupos de la muestra – PR, Brasil, 2025.

Grupo de muestra	N	Sexo (M/F)	Edad (grupo de edad)	Perfil
GM1 – Familiares de personas fallecidas	15	10F / 5M	22 - 70 años	Seis hijos e hijas, cinco cónyuges y el resto con vínculos cercanos (madre, nuera, sobrina, nieta). Nueve fallecimientos se produjeron en el hospital, uno en un centro de urgencias y cinco en el domicilio. Siete personas tenían seguro médico.
GM2 – Personas con cáncer avanzado	24	23F / 1M	25 - 64 años	15 vivían con su pareja; nueve recibían tratamiento a través del SUS y 14 en el sector privado o complementario. Cuatro PCA recibían seguimiento por parte de equipos multidisciplinares especializados en CP; ocho tenían DAV diagnosticadas.
GM3 – Médicos	20	12F / 8M	30 - 58 años	11 Oncólogos (dos cirujanos), nueve especialistas en cuidados paliativos (cuatro en programas domiciliarios). Cinco de ellos trabajaban en hospitales filantrópicos, siete en hospitales públicos y dos en privados. Cinco realizan cuidados paliativos domiciliarios.
GM4 – Psicólogos y trabajadores sociales	15	13F / 2M	25 - 72 años	11 psicólogos y cuatro trabajadores sociales. Cuatro de ellos trabajaban en hospitales, uno en un centro de cuidados paliativos, cuatro en servicios de atención domiciliaria, cinco en consultas y dos en una institución sin ánimo de lucro para personas con cáncer.
GM5 – Enfermeros, técnicos de enfermería y fisioterapeutas	22	17F / 5M	26 - 55 años	17 enfermeros, dos técnicos de enfermería y tres fisioterapeutas. De ellos, 14 trabajaban en el SUS en entornos hospitalarios (consultas externas, hospitalizaciones, UCI y cuidados paliativos) y cinco en UCC vinculadas a planes de salud y uno en un servicio privado de cuidados paliativos.
Total	96	75F / 21M	De 22 a 82 años	Diversidad de roles familiares y profesionales en diferentes entornos asistenciales.

Nota: F = femenino; M = masculino; CP = cuidados paliativos; SAMU = Servicio de Asistencia Móvil de Urgencias; DAV = Directivas Anticipadas de Voluntad; UPA = Unidad de Atención de Urgencias; UCC = Unidades de Cuidados Continuados; SUS = Sistema Único de Salud.

La diversidad geográfica e institucional permitió identificar cuatro escenarios de atención: Escenario I (SUS con equipo de cuidados paliativos): atención multiprofesional con asistencia domiciliaria ocasional; Escenario II (SUS sin cuidados paliativos estructurados): seguimiento mayoritariamente a cargo de oncólogos, con el apoyo de la Estrategia de Salud Familiar o del Programa “Melhor em Casa” (Mejor en Casa); Escenario III (plan con red propia y CP estructurado): equipo especializado, atención domiciliaria las 24 horas y suministro de medicamentos; Escenario IV (plan sin estructura de CP o atención privada): atención centrada en el oncólogo, con búsqueda autónoma de apoyo complementario.

El análisis de los datos de los cinco GM permitió la construcción de la teoría sustantiva denominada “La construcción social del lugar de muerte en el cáncer avanzado: entre mediaciones y obstáculos”, constituida por dos categorías: 1) Viabilizando elecciones y mediando significados: la comunicación como puente entre deseos y realidades y, 2) Barreras sistémicas: obstáculos para la construcción de significados compartidos. En este artículo se presentarán la primera categoría y sus subcategorías, a saber: 1) El lugar de muerte como espacio simbólico y 2) Dinámicas relacionales: conflicto, mediación y viabilización.

En la Figura 1 se observa la contraposición entre el hospital y el domicilio como opciones de “elección” para el lugar de muerte. Se identifica que no hay unanimidad en la elección; en realidad, el hospital, para muchos, representa tanto un sentimiento de seguridad, control y alivio del dolor como de frialdad y distanciamiento emocional; mientras que el domicilio transmite, en determinados momentos, una idea de respeto a la autonomía, la intimidad y el sentimiento de pertenencia, pero en otros momentos se considera generador de miedos e inseguridad. La toma de decisiones implica un proceso de negociación y consenso modulado por la ponderación de factores subjetivos (creencias, espiritualidad, recuerdos), institucionales (protocolos, cerco de silencio) y sistémicos (falta de recursos, condiciones clínicas, sobrecarga de los cuidadores, fallas de comunicación). Las flechas circulares evidencian la importancia del diálogo entre el paciente, los familiares y los profesionales de la salud para mediar y hacer viables las elecciones, que, aunque sean adecuadas en una situación determinada, pueden dejar de serlo en otra.

**Figura 1:** Diagrama que resume los procesos experimentados/identificados en la categoría – Maringá, PR, Brasil, 2025.

### Habilitando Elecciones y Mediando Significados: la Comunicación Como Puente Entre Deseos y Realidades

En esta categoría se observa que la comunicación desempeña un papel central en la mediación de significados, construyéndose a partir de las interacciones simbólicas entre el paciente, los familiares y los profesionales de la salud. Esta mediación permite la construcción de significados compartidos sobre el lugar de la muerte, con el objetivo de respetar la autonomía y preservar la dignidad hasta el final de la vida. En este contexto, se identificó que el manejo adecuado del dolor y el sufrimiento constituye el elemento simbólico central en la modulación, resignificación y definición de una nueva decisión, que puede tanto reafirmar la primera como modificarla.

En la Cuadro 3 se presenta un resumen de los comentarios y las subcategorías que integran la categoría y ejemplifican los hallazgos.

**Cuadro 3:** Categoría, subcategorías y declaraciones de los participantes – PR, Brasil, 2025.

Facilitar las elecciones y mediar en los significados: la comunicación como puente entre deseos y realidades	
*El lugar de la muerte como espacio simbólico*	*[...] puedo cambiar de opinión, pero, en principio, mi deseo es que sea en el hospital, bien atendida, incluso para evitar que ellos se agoten [...] porque tengo a los niños pequeños [...]. Así que, en el hospital hay médicos, hay equipo. Me siento más segura en el hospital*. (PCA01, 41 años) *Porque en el hospital tienen recursos que nosotros no tenemos. Así que cuando le sedaron, se quedó tranquilo, muy quieto, y luego, de madrugada, falleció*. (F09, 62 años, esposa) *[...] a menudo, el paciente y la familia se sienten más seguros en el hospital [...] al tener recursos más cercanos, como oxígeno y medicación de forma inmediata. A menudo ocurre que el contexto emocional es más vulnerable [...] ya sea porque los familiares no desean presenciar el fallecimiento o porque hay niños en casa, por lo que optan por que el paciente sea trasladado al hospital*. (MED-15, 34 años) *Quiero estar en casa con las personas que amo, en el lugar donde me siento bien, donde me siento acogida. Esta es mi petición*. (PCA15, 39 años) *Decidieron quedarse en casa, cerca de la familia, por el confort del hogar y la comodidad. Ya no había mucho que hacer*. (FA06, 33 años, hijo) *El ambiente hospitalario no es tan acogedor como estar en casa con los familiares más cercanos. Estar allí, en tu propio espacio, en tu rinconcito*. (PS08, 35 años) *Lo importante es no tener dolor, estar en un entorno más adecuado*. (PCA03, 45 años) *Pienso que al final de la vida es importante que te cuiden bien, porque el dolor no es agradable*. (PCA23, 48 años)
*Dinámicas relacionales: conflicto, mediación y viabilidad*	*Mis hijos y yo lo decidimos. Ellos decían: “Papá, no puedes dejarla aquí, tenemos que llevárnosla”; si moría allí, al menos recibiría atención médica*. (FA02, 69 años, esposo) *La insuficiencia respiratoria empezó a empeorar mucho, estaba casi agonizando. El marido llamó a los tres hijos y les dijo: “Habíamos prometido que no la dejaríamos morir en el hospital, que se quedaría aquí en casa con nosotros, pero viendo por lo que está pasando ahora, no puedo dejarla sufrir así. Si en el hospital le ponen al menos un catéter nasal para que respire hasta que se vaya, será mejor”. Entonces decidieron llevarla, así que lo hablaron entre ellos*. (FA05, 22 años, sobrina). *Hay situaciones en las que el paciente quiere irse a casa, pero la familia no quiere. Para poder atenderlo hay que turnarse, hay que invertir en profesionales. Entonces la familia dice: “No hay manera”, te vas a quedar aquí en el hospital*. (ENF17, 26 años) *Si la hubiéramos llevado al hospital, quizá alguien se habría quedado con ella o la habrían ingresado en la UCI y habría muerto allí sola. Ella tampoco quería ir, por eso decidimos pasar por ese calvario, porque fue un calvario. Mi hermana no podía... llegó incluso a llamar a la ambulancia. Yo soy un poco más tranquila y me quedé a su lado todo el tiempo. Le estaba cogiendo la mano en el momento en que murió. El médico de la ambulancia dijo: “Depende de ustedes, estamos aquí para hacer lo que quieran, solo que... la única diferencia es que aquí está rodeada de amor, y en el hospital se quedará allí..., pero si quieren, la llevamos”. Entonces decidimos que no la llevaríamos”*. (FA11, 44 años, hija)

### El Lugar de la Muerte Como Espacio Simbólico

La elección del hospital está relacionada con la seguridad técnica y tiene el significado de un espacio que ofrece un control eficaz del dolor y el sufrimiento, gracias a la disponibilidad inmediata de recursos y apoyo profesional. La elección del hospital también está influenciada por valores culturales, creencias religiosas, experiencias personales con los servicios de salud y vivencias relacionadas con la fase terminal. Por ejemplo, a partir de la preocupación por mitigar el sufrimiento de los familiares, principalmente de los niños al presenciar los momentos finales; el aumento de la sobrecarga familiar en los cuidados finales; e incluso evitar recuerdos negativos en el entorno doméstico, lo que hace que el hospital se convierta en el lugar de elección para muchos.

Por otro lado, el hogar se simboliza como un lugar de dignidad y comodidad y representa conexión emocional, acogida, intimidad, respeto a la autonomía del paciente en fase terminal y fortalecimiento de los lazos familiares. La búsqueda de dignidad y autonomía está intrínsecamente relacionada con el significado atribuido al dolor y al sufrimiento, siendo el manejo eficaz de estos factores el principal determinante en la toma de decisiones. Cuando el dolor y el sufrimiento no pueden controlarse adecuadamente, el hogar deja de ser una opción viable, lo que pone de manifiesto el distanciamiento del confort deseado.

Otro aspecto relevante es que el manejo adecuado del dolor simboliza dignidad y respeto en los momentos finales de la vida. Este manejo trasciende el control físico, alcanzando dimensiones emocionales y simbólicas, lo que influye directamente en la percepción de dignidad y en la viabilidad del lugar elegido para que ocurra la muerte. Cuando el control del dolor y el sufrimiento es inadecuado, la percepción de dignidad se ve comprometida, y el hospital se reinterpreta como un espacio de alivio y seguridad.

### Dinámicas Relacionales: Conflicto, Mediación y Viabilidad

La búsqueda del alivio o la privación del dolor y el sufrimiento desencadena un proceso de negociación sobre el lugar de la muerte, en el que participan las personas con cáncer, sus familiares y los profesionales de la salud. Esta dinámica, marcada por conflictos, negociaciones, mediaciones y viabilización, está impregnada de comunicación, que actúa como elemento central para alinear expectativas y facilitar la toma de decisiones.

Las dinámicas familiares ilustran el *self* colectivo, en el que los significados se negocian entre los miembros. Cabe señalar además que los conflictos en la toma de decisiones pueden surgir por divergencias entre los deseos de la persona con cáncer y los de sus familiares. En general, los conflictos surgen de diferentes interpretaciones sobre lo que es “mejor” para la persona con cáncer. Estos conflictos son momentos de negociación simbólica, en los que los significados se ajustan o reafirman mediante la mediación. Los agentes mediadores de este proceso de resignificación continua pueden ser los familiares o los profesionales de la salud.

El *self* de los familiares también se moldea en la interacción, al asumir roles de cuidado, negociación y toma de decisiones. El conflicto entre familiares puede interpretarse como una lucha simbólica por la preservación del *self* colectivo de los familiares. En estos casos, los profesionales de la salud desempeñan un papel crucial en la facilitación de esta mediación, siendo esenciales en la resignificación de miedos y expectativas al hacer posibles nuevas experiencias que puedan resignificar el lugar de la muerte, y ayudando así a los familiares a alinear los significados y las expectativas. Esta mediación no solo resuelve conflictos, sino que también crea significados compartidos sobre el lugar de la muerte.

## DISCUSIÓN

Los resultados evidencian la importancia de la mediación profesional, la escucha activa y la comunicación eficaz para favorecer la negociación de significados y decisiones compartidas entre pacientes, familiares y equipos de salud —aspectos identificados como fundamentales para la promoción de cuidados centrados en la persona y en la calidad de vida en la fase terminal. Estudios previos destacan que la comunicación asertiva y la participación de los profesionales de enfermería en el proceso de toma de decisiones son estrategias cruciales para garantizar la autonomía del paciente y fortalecer la relación terapéutica en el contexto de los cuidados paliativos, contribuyendo a prácticas más humanizadas y alineadas con los valores individuales de los pacientes^(19,20)^.

La categoría “Facilitando elecciones y mediando significados: la comunicación como puente entre deseos y realidades” muestra que la decisión sobre el lugar de muerte constituye un proceso simbólico, interactivo y relacional. Este estudio amplía la comprensión del fenómeno al evidenciar que símbolos como el hospital, el hogar, el dolor y la dignidad integran los significados atribuidos al lugar de muerte, los cuales se negocian constantemente y se hacen viables o inviables ante las condiciones estructurales y relacionales disponibles. Este conocimiento ofrece fundamentos teóricos para las prácticas y políticas en cuidados paliativos, lo cual se ve reforzado por investigaciones que demuestran que los elementos simbólicos orientan tanto a los familiares como a los equipos multiprofesionales en la co- construcción de estos significados^(21,22)^.

Los significados atribuidos al lugar de la muerte surgen de las relaciones sociales y se moldean por la manera en que los sujetos interpretan sus experiencias^(12)^. Así, el hospital se simboliza como un espacio de protección y atención técnica o, por el contrario, como un lugar de distanciamiento y ruptura con la dignidad, tal como se revela en este y otros estudios^(3,6)^. De este modo, el lugar de la muerte adquiere diversos significados según el repertorio simbólico que el paciente y sus interlocutores construyen a lo largo de la trayectoria de la enfermedad.

En contextos no oncológicos, también se identificaron resultados similares. Estudios internacionales^(6,8,23)^ señalan que, en enfermedades como la demencia o la insuficiencia renal crónica, el hospital tiende a ser el lugar predominante de muerte, pero en desacuerdo con las preferencias expresadas previamente, sobre todo debido al insuficiente apoyo domiciliario y a la sobrecarga familiar. Esto refuerza que la construcción social del lugar de muerte —a veces como espacio de dignidad y vínculo, otras como escenario de ruptura e institucionalización— no es exclusiva del paciente con cáncer, sino que atraviesa diferentes procesos de enfermedad.

En Brasil, los estudios^(7,24)^ indican el predominio del hospital como lugar de muerte para las personas con cáncer, sobre todo ante el empeoramiento clínico o la falta de apoyo domiciliario^(7)^. En el presente estudio, también significó protección familiar, especialmente para evitar que los niños presenciaran la terminalidad, previniendo impactos emocionales y aliviando la sobrecarga de los seres queridos. Por otro lado, el hogar simbolizaba el respeto a la dignidad y la autonomía, además del mantenimiento de la conexión emocional, proporcionada por la proximidad con los seres queridos^(6)^ y la preservación del *self*. Sin embargo, cuando los familiares no se sienten preparados para afrontar la evolución natural de los cuidados al final de la vida y comienzan a experimentar un gran sufrimiento, esta preferencia puede adquirir un nuevo significado, convirtiendo al hospital en una opción inevitable^(5)^. Por lo tanto, la muerte en casa representa no solo una elección de lugar, sino una afirmación del *yo* en su entorno afectivo, lo que demuestra que la geografía del cuidado es, ante todo, una narrativa de identidad.

No obstante, hay que considerar que las diferencias regionales en la disponibilidad de servicios especializados y la dificultad de acceso pueden influir en la elección del lugar de muerte. Además, según algunos informes, en el contexto del cáncer, las diferencias en el acceso y la disponibilidad de servicios son notables, incluso dentro de la misma región o estado, dependiendo del lugar de residencia —ya sea la capital o el interior, o una zona urbana o rural—. Este hecho tiene sin duda implicaciones importantes para la equidad en los cuidados paliativos. Un estudio sobre la tendencia temporal de la mortalidad por neoplasia en Brasil entre 2002 y 2022 constató, por ejemplo, que São Paulo y Rio Grande do Sul presentaron altas tasas de fallecimientos en el entorno hospitalario, posiblemente asociadas a una mayor oferta de servicios de referencia oncológica. Por otro lado, los estados del Norte y el Nordeste, como Maranhão y Piauí, registraron tendencias crecientes de fallecimientos en el domicilio, lo que puede estar relacionado con la baja cobertura de servicios especializados^(24)^.

A pesar de estas cuestiones, las evidencias indican que las políticas públicas sólidas, que garantizan el acceso, la escucha y el apoyo continuo, aumentan la posibilidad de alinear el lugar de la muerte con las preferencias del paciente, promoviendo la dignidad, el confort y la reducción de intervenciones innecesarias al final de la vida^(25,26)^. De este modo, la PNCP instituida en Brasil en 2024^(10)^ representa un avance importante para superar las barreras estructurales, ampliando el acceso a la atención domiciliaria, fortaleciendo equipos multiprofesionales y alineándose con modelos internacionales exitosos^(26)^. Para ello, será necesario que la Atención Primaria de Salud se reestructure con equipos de cuidados paliativos para que los pacientes y sus familiares puedan contar con apoyo en el manejo del dolor y el sufrimiento, evitando así la necesidad de acudir al hospital en el momento de la agudización del sufrimiento^(27)^.

La PNPC tiene como objetivo el desarrollo de acciones que estimulen la educación en cuidados paliativos, incentivando la formación y la educación continua de los profesionales de los equipos que actúan en la Red de Atención de Salud^(28)^, en la que el equipo de enfermería tiene un papel preponderante en la integración entre los demás miembros del equipo y la unidad de cuidados del paciente y sus familiares.

Además, la construcción del *self* en el proceso de terminalidad, entendida como la forma en que la persona se ve a sí misma y es reconocida por los demás^(29)^, se moviliza constantemente. El deseo de permanecer en casa expresa la intención de mantener la continuidad del *yo* ante la finitud; mientras que la hospitalización, aunque restringe el control, puede simbolizar protección y esperanza de amparo ante el sufrimiento. Esto reitera que las preferencias oscilan según la negociación entre los valores personales y las necesidades clínicas. La mediación profesional surge como un elemento clave en la reconstrucción de significados. Los profesionales que escuchan y acogen amplían las posibilidades al actuar como mediadores simbólicos del proceso de morir^(29)^. Al asumir el papel del otro^(30)^, estos profesionales favorecen la expresión del *self* y contribuyen a una decisión compartida, anclada en valores y deseos reales. De este modo, los conflictos familiares surgieron como un aspecto en el proceso de toma de decisiones sobre el lugar de la muerte y resaltan la importancia del papel del enfermero no solo como cuidador clínico, sino también como mediador comunicacional entre los miembros de la familia.

La literatura sobre cuidados paliativos destaca que los profesionales de enfermería pueden poner en práctica técnicas de mediación familiar mediante estrategias como reuniones familiares estructuradas, el uso de una comunicación empática y facilitadora, la escucha activa y la orientación para la resolución de divergencias de valores y expectativas, promoviendo un entendimiento compartido y decisiones conjuntas sobre la atención^(31)^. Estos enfoques pueden contribuir a reducir tensiones, fortalecer el apoyo emocional y alinear las decisiones sobre el lugar de la muerte con las preferencias de los pacientes y sus familiares, integrando habilidades clínicas y comunicativas en el cuidado holístico.

Además, es necesario reconocer las limitaciones marcadas por conflictos familiares insolubles, estructuras precarias y resistencia institucional a la escucha activa, que constituyen límites éticos y contextuales que tensan esta mediación e impactan en la viabilidad del lugar de muerte deseado, momento en el que cabe la realización de una conferencia familiar, como instrumento terapéutico en pro de la resolución de conflictos y dilemas éticos^(32)^.

En este contexto, la conferencia familiar es una intervención terapéutica, mediada por profesionales del equipo de cuidados paliativos, que constituye un espacio estructurado de comunicación que favorece la escucha activa, la expresión de valores y la negociación de expectativas entre pacientes, familiares y el equipo de salud, reduciendo conflictos y promoviendo decisiones compartidas. Al actuar como mediador comunicacional, el profesional, que en muchos casos es el enfermero, contribuye a alinear las decisiones sobre la atención —incluido el lugar de la muerte— con las preferencias del paciente, fortaleciendo el entendimiento mutuo y la atención centrada en la persona^(32)^.

La interacción entre pacientes, familiares y profesionales de la salud puede desencadenar conflictos simbólicos que exigen una comunicación eficaz y la búsqueda de consenso. En contextos como el de China, el “cerco del silencio” -la ocultación de la condición para la protección emocional - limita la autonomía y dificulta las decisiones compartidas^(33)^, y se reproducen escenarios similares en Brasil, agravados por la fragmentación de los servicios y la ausencia de política^(7)^.

Además, las instituciones de salud enfrentan desafíos en la comunicación sobre el proceso de morir y la muerte, ya que los modelos biomédicos aún predominan y el lenguaje técnico con frecuencia oscurece la escucha y la expresión de los deseos del paciente^(9,34)^. En este entorno, el “cerco del silencio”, aunque culturalmente motivado, perjudica la participación activa del paciente en el proceso de toma de decisiones^(30,33)^, rompiendo la construcción compartida de significados e impidiendo que el *yo* se exprese ante la finitud.

La interacción institucional reduce la muerte a un evento técnico, negando su dimensión simbólica, afectiva y relacional. Además, no solo oscurecen o distorsionan los significados atribuidos por el paciente en estado terminal y sus familiares, sino que también contribuyen a la ausencia de una planificación previa adecuada para los momentos finales de la vida. Por lo tanto, la comunicación clara y empática entre profesionales, pacientes y familiares es fundamental para mitigar las barreras y facilitar las decisiones^(5,8)^. Sin embargo, la mediación profesional, aunque deseable y potente, depende de la estructura, el tiempo, la preparación emocional y la legitimidad institucional.

Ante este desafío comunicacional, el enfermero —como mediador simbólico y profesional de referencia en el cuidado cotidiano— debe perfeccionar sus habilidades de escucha terapéutica y comunicación segura. Para ello, debe saber identificar el momento oportuno para abordar las dudas sobre el proceso de morir y la muerte con el paciente y sus familiares^(35)^, a fin de restablecer la interacción horizontal y la co-construcción de significados en el proceso de toma de decisiones.

La Resolución n.º 564/2017 del Código de Ética de los Profesionales de Enfermería destaca la responsabilidad del enfermero de promover condiciones para la autonomía del paciente en la toma de decisiones sobre cuidados y confort^(36)^. En el ámbito internacional, el Código de Ética del Consejo Internacional de Enfermeras^(37)^ también refuerza el respeto a la dignidad humana, los derechos y las elecciones de los pacientes en todas las etapas de la vida, incluido el final de la vida.

Estos referentes éticos, tanto nacionales como internacionales, evidencian que la práctica de la enfermería debe regirse por una actuación humanizada, sensible a la singularidad de cada persona y promotora de la autonomía, independientemente del lugar de atención^(38)^. En este contexto, el equipo de enfermería se configura como mediador simbólico, ya que la elección del lugar de muerte exige no solo infraestructura, sino también relaciones horizontales, escucha activa y ambientes simbólicamente acogedores, lo que permite la realización de acciones personalizadas^(20)^ que atiendan las necesidades específicas y valoricen el “hoy” con sus limitaciones y posibilidades, promoviendo la calidad de los días que quedan.

El reconocimiento de que la elección del lugar de muerte es una construcción simbólica exige que, además de la infraestructura, se garantice a las personas el poder de atribuir sentido a esa decisión mediante interacciones horizontales y entornos acogedores^(5)^. Los modelos de atención que no tienen en cuenta el lenguaje, los valores y las dinámicas sociales tienden a reproducir desigualdades y prácticas deshumanizantes, mientras que un enfoque centrado en los significados puede fortalecer la eficacia clínica, la justicia social y el cuidado ético al final de la vida. Por lo tanto, reconocer el carácter relacional y simbólico de estas decisiones orienta prácticas y políticas que valorizan el protagonismo de los pacientes, promueven la equidad y permiten una asistencia verdaderamente humanizada en los cuidados paliativos.

Entre las posibles limitaciones del estudio se citan las entrevistas retrospectivas con familiares, que pueden implicar reconstrucciones subjetivas de los acontecimientos finales. Además, el predominio de entrevistas a distancia -que, por un lado, ampliaron el alcance geográfico - puede haber limitado la percepción de matices no verbales. Por último, la concentración de participantes procedentes de zonas urbanas y de género femenino no permitió captar las realidades rurales y las diferentes perspectivas de género. Así, considerando que los significados atribuidos al lugar de muerte se negocian y se hacen viables o inviables ante las diferentes condiciones estructurales y relacionales, se cree que la valoración de los matices del contexto urbano y rural y la participación de un mayor número de hombres, sobre todo entre los pacientes en cuidados paliativos y sus familiares, puede enriquecer la comprensión del fenómeno.

Se señala, por lo tanto, la necesidad de futuras investigaciones en diversos contextos asistenciales.

## CONCLUSIÓN

La teoría sustantiva construida describe la decisión sobre el lugar de muerte como un proceso simbólico, interactivo y dinámico. El análisis de las narrativas reveló capas de significados negociados en torno al hospital y al domicilio, influenciados por valores culturales, experiencias previas, condiciones institucionales y recursos disponibles. Este enfoque permitió comprender cómo se construyen, resignifican y comparten estos significados, lo que permitió identificar factores que moldean esta decisión en el contexto oncológico.

La elección del lugar de muerte se configura como una expresión del *yo* mediada por el lenguaje, la escucha activa y la capacidad de asumir el papel del otro. La interdependencia entre el paciente, los familiares y el equipo multiprofesional —en especial el papel del enfermero como mediador simbólico— es fundamental para garantizar tiempo, acogida y dignidad. Las implicaciones para la práctica y las políticas públicas incluyen la inversión en cuidados domiciliarios, respaldados por la PNCP. Futuros estudios longitudinales deben comparar las realidades antes y después de la implementación de la PNCP para evaluar su impacto en la viabilización de las elecciones, la mediación de significados y la superación de barreras sistémicas.

## Data Availability

El conjunto de datos que respaldan las conclusiones de este estudio está disponible previa solicitud al autor correspondiente.
